# The mechanistic functional landscape of retinitis pigmentosa: a machine learning-driven approach to therapeutic target discovery

**DOI:** 10.1186/s12967-024-04911-7

**Published:** 2024-02-06

**Authors:** Marina Esteban-Medina, Carlos Loucera, Kinza Rian, Sheyla Velasco, Lorena Olivares-González, Regina Rodrigo, Joaquin Dopazo, Maria Peña-Chilet

**Affiliations:** 1 Andalusian Platform for Computational Medicine, Andalusian Public Foundation Progress and Health-FPS, Seville, Spain; 2 Systems and Computational Medicine Group, Institute of Biomedicine of Seville, IBiS, University Hospital Virgen del Rocío/CSIC/University of Seville, 41013 Seville, Spain; 3 Group of Pathophysiology and Therapies for Vision Disorders, Príncipe Felipe Research Center (CIPF), 46012 Valencia, Spain; 4 Biomedical Research Networking Center in Rare Diseases (CIBERER), Health Institute Carlos III, 28029 Madrid, Spain; 5 Department of Physiology, University of Valencia (UV), 46100 Burjassot, Spain; 6 Department of Anatomy and Physiology, Catholic University of Valencia San Vicente Mártir, 46001 Valencia, Spain; 7 Joint Research Unit on Endocrinology, Nutrition and Clinical Dietetics UV-IIS La Fe, 46026 Valencia, Spain; 8 BigData, AI, Biostatistics & Bioinformatics Platform, Health Research Institute La Fe (IISLaFe), 46026 Valencia, Spain

**Keywords:** Retinitis pigmentosa, Rare diseases, Drug-repurposing, Disease maps, GABAergic neurotransmission, Systems medicine, Target-discovery

## Abstract

**Background:**

Retinitis pigmentosa is the prevailing genetic cause of blindness in developed nations with no effective treatments. In the pursuit of unraveling the intricate dynamics underlying this complex disease, mechanistic models emerge as a tool of proven efficiency rooted in systems biology, to elucidate the interplay between RP genes and their mechanisms. The integration of mechanistic models and drug-target interactions under the umbrella of machine learning methodologies provides a multifaceted approach that can boost the discovery of novel therapeutic targets, facilitating further drug repurposing in RP.

**Methods:**

By mapping Retinitis Pigmentosa-related genes (obtained from Orphanet, OMIM and HPO databases) onto KEGG signaling pathways, a collection of signaling functional circuits encompassing Retinitis Pigmentosa molecular mechanisms was defined. Next, a mechanistic model of the so-defined disease map, where the effects of interventions can be simulated, was built. Then, an explainable multi-output random forest regressor was trained using normal tissue transcriptomic data to learn causal connections between targets of approved drugs from DrugBank and the functional circuits of the mechanistic disease map. Selected target genes involvement were validated on *rd10* mice, a murine model of Retinitis Pigmentosa.

**Results:**

A mechanistic functional map of Retinitis Pigmentosa was constructed resulting in 226 functional circuits belonging to 40 KEGG signaling pathways. The method predicted 109 targets of approved drugs in use with a potential effect over circuits corresponding to nine hallmarks identified. Five of those targets were selected and experimentally validated in *rd10* mice: *Gabre*, *Gabra1* (GABARα1 protein), *Slc12a5* (KCC2 protein), *Grin1* (NR1 protein) *and Glr2a*. As a result, we provide a resource to evaluate the potential impact of drug target genes in Retinitis Pigmentosa.

**Conclusions:**

The possibility of building actionable disease models in combination with machine learning algorithms to learn causal drug-disease interactions opens new avenues for boosting drug discovery. Such mechanistically-based hypotheses can guide and accelerate the experimental validations prioritizing drug target candidates. In this work, a mechanistic model describing the functional disease map of Retinitis Pigmentosa was developed, identifying five promising therapeutic candidates targeted by approved drug. Further experimental validation will demonstrate the efficiency of this approach for a systematic application to other rare diseases.

**Supplementary Information:**

The online version contains supplementary material available at 10.1186/s12967-024-04911-7.

## Background

Retinitis pigmentosa (RP) is a rare hereditary disease that mainly affects the photoreceptors in the retina, resulting in progressive vision loss. RP is typically diagnosed in childhood and rather than a single entity, RP is better described by a group of disorders characterized by night blindness, narrowing of the visual field, and ultimately, legal blindness [1]. Due to RP’s relatively high prevalence among rare diseases, affecting more than 1.5 million patients worldwide (from 1/2500 to 1/7000) [2], it is considered the most common inherited disease of the retina [3]. It is a complex disorder with multiple genetic causes and diverse clinical manifestations, counting mutations in over a hundred genes known to lead to the development of the disease [4]. However, the genetic cause of RP remains unknown in 50% of the patients [5]. In addition, current therapeutic options for RP are limited, with only a few treatments available that can slow down the progression of the disease or improve the quality of life of patients.

Increasing the knowledge of the molecular basis of the disease is a crucial step in the search for new therapeutic options to treat RP. The exponential growth of genomic data, fueled by sequencing technologies [6], has revolutionized the field of rare diseases with foreseeable achievements such as being able to diagnose all Mendelian diseases in the near future [7]. The knowledge generated in the last decade has expanded by more than two orders of magnitude the number of variants with known phenotypic effects [8]. However, the study of diseases has undergone a conceptual shift, moving from a focus on individual genes to exploring how they interact within cells [9]. This shift has led to the use of network-based models that rely on accumulated biological knowledge to identify causal relationships between genes, as defined by biological pathways [10]. The so-called “mechanistic models of human diseases” were developed by combining these novel approaches with biological human data.

Mechanistic models can be defined as mathematical representations of biological systems that aim to capture the underlying mechanisms that drive the behavior of these systems. Interestingly, these models can provide a causal link between gene expression and functional cell behavior [11]. However, the real potential of mechanistic models relies on their capability to reduce the complexity of the data while reinforcing interpretative power, unveiling specific interactions and functional outcomes [10]. Indeed, mechanistic models have helped to understand the disease mechanisms behind different cancers [12–14], rare diseases [15], complex diseases such as diabetes [15], or obesity [16], as well as the mechanisms of action of drugs [17], including drug repurposing [18] or gender-specific effects of drugs in cancer [19]. A relevant property of mechanistic models is that they model causality in a quite accurate manner [9]. Therefore, they can predict the potential consequences of gene perturbations (e.g. knock-out, drug inhibition effect, etc.) over pathway activities, as well as the downstream functional consequences in the cell [10, 20]. They have been used to predict candidate essential genes in cancer cell lines, whose further inhibition validated the predictions [12]. Repurposable drug candidates were proposed for Fanconi Anemia [17] which were further experimentally validated [21], and for COVID-19 [18], also validated using a cohort of around 16,000 patients of the Andalusian healthcare database [22, 23]. These computational methods represent a leap in the generation and testing of hypotheses guided by a specific functional rationale, especially useful in scenarios with limited knowledge like rare diseases such as RP.

The development of novel therapies is a slow and costly process, particularly in rare diseases, where orphan drug research accounts for limited resources. Drug repurposing, the process of discovering new therapeutic uses for existing drugs [24], emerges as a potential solution to this problem in this context [25], reducing the time and expenses involved in drug development [26].

In this work, we propose a data-driven approach to construct actionable models that describe the disease mechanism of RP. Moreover, by making use of explainable machine learning models, trained with publicly available gene expression data, we have estimated the impact that targets of drugs already in use have in the signaling mechanisms of RP, allowing us to infer and select the most suitable therapeutic target genes that will lead us to drug candidates for its repurposing in RP. Indeed, the role in RP progression of six of the proposed targets has been experimentally validated. The comprehensive analysis of the results enabled us to identify drug targets that hold significant potential in impacting disease mechanisms able to revert cell functional processes to a healthy state.

## Methods

**Fig. 1:**
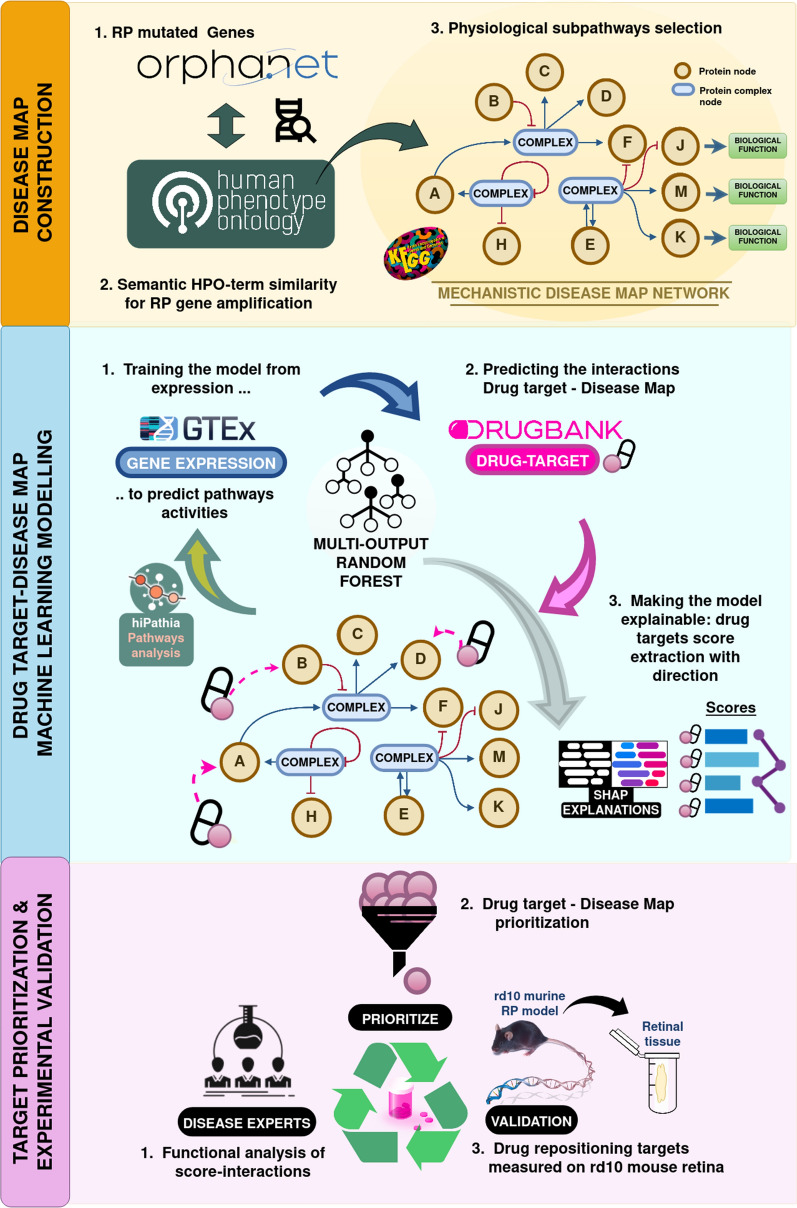
Diagram of the steps involved in the machine-learning-based drug repositioning modelization of Retinitis Pigmentosa (RP). RP Disease Map construction (yellow block); machine learning modelization of drug-targets over the RP map (blue block); target prioritization for experimental validation (pink block)

The overall methodology developed for RP modeling is divided into three main stages depicted in Fig. 1. Briefly, the process entails collecting data on RP-related mechanisms from existing open-access databases (DBs). We ensured the precision and fidelity of RP-phenotypes and RP-gene associations by using standardized definitions from OrphaNet [27], OMIM [28], and the Human Phenotype Ontology DBs [29]. The information retrieved, together with the accumulated knowledge on functional gene interactions provided by the KEGG (Kyoto Encyclopedia of Genes and Genomes) pathways DB [30], is used to produce a map of functional interactions among RP-associated genes and genes upstream and downstream in the pathways, thus defining a set of sub-pathways, the so-called signaling circuits, which can be considered a reasonable representation of the RP disease map [17, 18]. This RP mechanistic map can be used to build a signal transduction model that allows the estimation of RP-related cell functions from gene activities measured as gene expression levels [12]. The model of the RP mechanistic map is then integrated into an explainable machine learning (ML) model, which infers potentially causal relationships between genes of interest, in our case known drug targets (KDTs) extracted from the DrugBank DB, and the RP-related functional activities as described in the RP mechanistic map. The ML methodology prioritizes KDTs with a high predictive score, degranulating the results into the specific influences that each KDT has over the different parts of the mechanistic map. The results highlight KDTs that are predicted as relevant in regulating the functional landscape of RP mechanistic map and could potentially be further studied as promising therapeutic targets.

### Gene expression data

Transcriptomic data from the GTEx (Genotype-Tissue Expression) Portal was used to obtain meaningful interactions among proteins and the role of these proteins in the modulation of the signaling pathways. Data from 54 non-diseased tissue sites across 948 individuals, with a total of 17,382 samples over 22,000 gene expression values were downloaded from the GTEx Portal (GTEx Analysis Release V8; dbGaP Accession phs000424.v8.p2) [31]. Gene expression data from the RNA-seq GTEx dataset was normalized by applying the Trimmed mean of M values (TMM) normalization using the edgeR R/Bioconductor package [32].

### Data on drug targets

To investigate the potential effect druggable molecules have over our RP-mechanistic model, the DrugBank DB release 5.1.8 [33] was used. Drug-target interactions involving human protein-target of approved drugs with a known pharmacological action were extracted. Only KDTs with gene expression measurements in the GTEx V8 dataset [31] were taken into account.

### Defining the core knowledge for the mechanistic map of retinitis pigmentosa

We define the mechanistic disease map as the collection of functional and directed interaction among genes, gathered in the physiological signaling pathways, that best resembles the disease mechanism. Therefore, all the knowledge available on RP-related genes must be collected to be set in the context of functional interactions provided by the KEGG pathways. To construct the RP mechanistic map, as a first step, we extracted the genes with mutations known to be related to the development of RP from OrphaNet [27] (ORPHA:791) and OMIM [28] databases.

### Expanding the mechanistic map of retinitis pigmentosa

To expand the core map, other genes linked to RP phenotypes that could be playing a key role in RP progression mechanisms were added from the Human Phenotype Ontology (HPO) database [29]. To do so, we extracted the phenotype-disease and phenotype-gene interactions from HPO phenotypic abnormality sub-ontology (hp.obo release 2019-09-06). A total of 8822 HPO-terms tagging a total of 5440 genes were assessed in this study. Specificity levels were assigned to the HPO-terms based on the hierarchical properties of the DB. Since the HPO ontologies have a hierarchical structure, the specificity level was calculated as the number of ancestor nodes an HPO-term has. Low-specificity HPO-terms can be very broad with many related processes and genes while high-specificity HPO-terms can describe very isolated ones with few connections. Only HPO-terms with a specificity level between 7 and 15, with traceable author statement (TAS) evidence sources were taken into account.

### Defining the mechanistic map of retinitis pigmentosa

This set of RP-related genes (extracted from both HPO and OrphaNet/OMIM databases) was mapped to the KEGG signaling pathways using the Hipathia R Bioconductor package [34]. Then, the receptor-effector sub-pathways, also known as circuits, containing the RP-genes were extracted. The collection of circuits containing the selected RP-related genes is what we define as the RP Mechanistic Map, an actionable *in-silico* model where simulations of different scenarios can be performed. Those pathways with a high number of disconnected nodes or which circuits have a maximum number of nodes of three were ruled out to minimise the potential errors in signal propagation algorithm estimation of Hipathia. The complete RP mechanistic map model can be explored at: http://hipathia.babelomics.org/RP_Mechanistic_Map/ [35].

### Modeling of the mechanistic map of retinitis pigmentosa

Mechanistic models provide a powerful framework for understanding the complex relationships between different components within a biological system. In this context, we used the mechanistic pathway analysis (MPA) tool Hipathia to model RP functional landscape. Hipathia is based on a recursive signal propagation algorithm that considers biological pathways as a collection of “signaling circuits”, graphs representing the interaction between proteins where the signal propagates from receptor-proteins to effector-proteins in a single path. Hipathia uses gene expression values as proxies of the level of activation of the corresponding protein node in each circuit. Taking into account the inferred protein activity and the interactions between the proteins (activations or inhibitions) defined in the pathway, the level of activity of each circuit is estimated from gene expression values using the following recursive equation.

$$S_{n} = v_{n} \cdot \left( {1 - \prod\limits_{{S_{a} \in A}} {\left( {1 - S_{a} } \right)} } \right) \cdot \prod\limits_{{S_{i} \in I}} {\left( {1 - S_{i} } \right)}$$

*S*_*n*_ being the signal intensity for the current node *n*; *v*_*n*_ being its normalized gene expression value; *A* being the set of activating signals (*s*_*a*_) arriving at the current node from activation edges; *I* being the set of inhibiting signals (*s*_*i*_) coming to the node from inhibition edges. A detailed explanation of the method can be found in *Hidalgo and cols 2017* [12].

The Hipathia method, as implemented in the Hipathia R/Bioconductor package [36], was used to estimate signaling circuit activities within the 79 physiological signaling KEGG pathways from the corresponding GTEx normalized gene expression values (rescaled to [0, 1] as recommended by the package documentation).

### Explainable machine learning model

This method employs a Multi-output Random Forest [37] and SHapley Additive exPlanations (SHAP) [38], tailored for tree-based algorithms [39], to effectively predict the signaling activity across a disease map based on the expression measures of key drug targets (KDTs). Simultaneously, it identifies the most relevant genes for accurately predicting each circuit.

Leveraging the SHAP rankings and the learner’s predictive prowess, the model implements a selection procedure to pinpoint potential drug targets (KDTs) that could regulate the disease map. This process is validated using the bootstrapping Nogueira procedure [40], which evaluates both the stability of the KDT selection method and the quality of the predictions.

The method culminates in the generation of circuit-specific confidence intervals and user-friendly thresholds for both measures, along with the identified KDTs and their associated relevance scores. For further details, please refer to Additional file 15: Methods.

### Target functional prioritization

Results from the machine learning model were rescaled to a [− 1, 1] range and plotted as a heatmap using the NMF R package [41]. Calculation of the best number of KDT clusters to select was done with the Gap Statistics method (Gap Statistics chooses the number of K, where the biggest jump in within-cluster distance occurred). Then, based on the generated hierarchical clustering on the Heatmap, KDTs were divided into three groups.

Enrichment analysis of GO (Gene Ontology) Molecular Function and Biological Processes of the KDTs belonging to each of the 3 groups was done using Panther DB and GO enrichment tool [42], those terms with adjusted p-value < 0.05 were considered significant.

The circuits integrating the RP Disease Map were functionally annotated with the UniProtKB DB (accessed on 10.09.2022). From the resulting annotations, and according to the molecular pathophysiology of RP [43, 44], 9 processes were identified, grouping the functions into RP hallmarks: Apoptosis, Necrosis, Stress Response, Inflammatory response, DNA integrity, Fatty acids, and lipid metabolism, Sensory and stimuli response, Development processes, Neuronal Processes. The RP disease map circuits were arranged and functionally tagged based on these RP hallmarks, resulting in nine functional modules. Functional profiling of relevant KDTs was performed to evaluate the impact of these KDTs on the nine RP hallmarks. Since circuits are labelled with specific functions that fall under these 9 RP hallmarks, our machine learning model predicts the most relevant KDTs for each circuit. Consequently, the effects of these KDTs on the nine RP hallmarks were determined using the corresponding circuits as proxies.

Relevant KDTs were selected for experimental analyses in RP rd10 murine model based on their relevance score (belonging to the top 10th percentile), their involvement in defined functional hallmarks and in interesting pathways with RP biological and clinical relevance.

The characteristics of the drugs targeting relevant KDTs were studied according to the Anatomical Therapeutic Chemical Classification (ATC) from the World Health Organization. Fisher’s exact test was used, against the Drugbank 5.1.8 DB, to search for over-represented drug categories from general (ATC level 1 with 14 main categories) to more specific terms (ATC level 4 with 918 categories). The ATC levels 1, 2, 3, and 4 were taken into account. ATC level 5 was not considered since it is composed of the names of the compounds and does not provide any kind of grouping.

### Animal model of retinitis pigmentosa

*Rd10* mice were used as a model for autosomal recessive RP. These mice have a mutation in the *Pde6b* gene, affecting rod photoreceptor function and leading to their degeneration. In our conditions, two peaks of photoreceptor degeneration were observed at postnatal days (P)18 and P60. C57Bl/6J mice, sharing the same genetic background, served as controls. Mice were housed under specific conditions at the Research Center Principe Felipe (CIPF) and treated according to ethical guidelines (European Union Directive (2010/63/EU). Each study type (gene expression, GABA content, western blot and retinal histological quantification) involved at least eight mice per group except for histological analysis. For further details, check Additional file 15: Methods. The procedure was approved by the Committee of Ethics in Research of CIPF.

### Isolation of total RNA and quantitative real-time PCR

Total RNA was isolated from frozen retinas of control and *rd10* mice aged P15 to P60 (eight retinas for each group) using the NZY Total RNA Isolation Kit, following the manufacturer’s protocol. After determining RNA concentration and synthesizing cDNA, we measured the relative expression of specific genes in retinas using real-time PCR. Expression was normalized using a housekeeping gene, and then further normalized to control mice values. For further details, and a list of probes used, check Additional file 15: Methods.

### Western blot

Retinas from control and *rd10* mice were homogenized and resuspended in 100 μL of RIPA buffer and boiled for 5 min. Electrophoresis was carried out on 8% SDS polyacrylamide denaturing gel at 25 mA for 2 h. Samples were then transferred to a PVDF membrane and incubated with primary antibodies against GABARɑ1, KCC2 and NR1 and subsequently with secondary antibodies. Protein bands were detected using NZY Advanced ECL and quantified using the Alliance Q9 Advanced. Western blots were quantified and normalized with β-tubulin using AlphaImager 2200 (alpha innotec, Germany). Detailed information in methodology and materials used for western blot assays can be found in Additional file 15: Methods.

### Retinal histology and quantification

Eyes from mice at P23 were processed for histological analysis. After fixation and cryosectioning, sections were permeabilized with 0.1% Triton X-100 for 1 h, incubated with anti-GABARɑ1, KCC2 and NR1 primary antibodies and fluorescence-conjugated secondary antibodies. After labelling and counterstaining with DAPI, the sections were mounted in Fluoromount-Gmounted. Confocal microscopy was used for observation of retinal inner nuclear layer (INL), retinal inner plexiform layer (IPL), retinal outer nuclear layer (ONL), retinal outer plexiform layer (OPL) and retinal ganglion cell layer (GCL). To confirm photoreceptor degeneration the number of nuclei at the outer nuclear layer (ONL) was measured. For further details, check Additional file 15: Methods.

### Determination of GABA in retinal extracts with liquid chromatography–mass spectrometry (HPLC–MS)

GABA concentrations in retinal extracts of control and *rd10* mice at P18 and P23 were measured using HPLC–MS. After homogenization, protein precipitation, and centrifugation, samples were loaded for LC–MS. Chromatography was performed, and the HPLC was coupled to a mass spectrometer. GABA concentration was determined using a standard curve and expressed in nmol/mg protein. Check Additional file 15: Methods for further details.

### Statistical analysis for preclinical validation

Statistical analysis was performed using GraphPad Software 9.0 (Prism; GraphPad Software, Inc, San Diego, CA). Normal distribution of data was analyzed by Shapiro–Wilk and Kolmogorov–Smirnov tests. Comparisons between age-matched control and *rd10* mice were performed using unpaired t-tests or Mann–Whitney test. A P value < 0.05 was considered statistically significant. The data were plotted using Graph Pad Software 9.0. The data were presented as mean ± SEM.

### Selection of drugs targeting candidate genes

Drugs targeting experimentally validated KDTs were filtered out based on pharmacological actions that counteracts the dysregulation observed in *rd10* model, which means that have the opposite effect (agonist/antagonist) on target that the observed in the RP model, both in target gene expression and protein quantification. Only drugs with potential to revert the RP dysregulation were further studied. This information was obtained from DrugBank database. Assessment of the resulting drugs over the RP hallmarks modules was carried out by measuring the percentage of the hallmark (no. of circuits from total no. of circuits of a given hallmark module) that was affected by each drug, taking the relevant KDTs that each drug targets as a proxy.

## Results

### Mechanistic disease map of retinitis pigmentosa

In this study, we present the mechanistic map for the study of the rare disease Retinitis Pigmentosa (RP). As a starting point, we retrieved from OrphaNet a list of 93 genes associated with RP. The gene Entrez IDs plus Gene symbols as well as the database identifiers are shown in Table 1. Since HPO is a DB that uses phenotype terms to tag genes and diseases, we have exploited this dual property to screen for the genes that share at least 10 out of 22 Retinitis Pigmentosa HPO-terms (RP-HPO), resulting in a total of 22 RP-HPO-terms, as shown in Table 2. In Additional file 11: Fig. S1A, we can see the number of genes sharing RP-HPOs added that were represented in KEGG signaling pathways. The gene symbols of the added genes per number of shared HPO-terms can be accessed in Additional file 1: Table S1 and Additional file 11: Fig. S1A.

**Table 1:** List of OMIM/ORPHA genes associated to RP with their corresponding Entrez ID and gene symbol

Disease ID	Entrez ID	Gene symbol	Disease ID	Entrez ID	Gene symbol
OMIM:615565	4751	**NEK2**	OMIM:616394	26160	**IFT172**
OMIM:613341	9227	**LRAT**	OMIM:618173	403	**ARL3**
OMIM:615233	5949	**RBP3**	OMIM:613862	10461	**MERTK**
OMIM:613731	6010	**RHO**	OMIM:617763	23404	**EXOSC2**
OMIM:613767	1258	**CNGB1**	OMIM:615922	9128	**PRPF4**
OMIM:617781	9742	**IFT140**	OMIM:312600	6102	**RP2**
OMIM:613809	7399	**USH2A**	OMIM:613581	50939	**IMPG2**
OMIM:617023	60509	**AGBL5**	OMIM:609033	28982	**FLVCR1**
OMIM:613794	6121	**RPE65**	OMIM:613582	5148	**PDE6G**
OMIM:616544	138050	**HGSNAT**	OMIM:300029	6103	**RPGR**
OMIM:180100	6101	**RP1**	OMIM:613464	123016	**TTC8**
OMIM:615725	57709	**SLC7A14**	OMIM:604232	55812	**SPATA7**
OMIM:613801	5158	**PDE6B**	OMIM:180105	3614	**IMPDH1**
OMIM:612674	26090	**ABHD12**	OMIM:600132	7287	**TULP1**
OMIM:617871	112752	**IFT43**	OMIM:609923	10210	**TOPORS**
OMIM:608380	375298	**CERKL**	OMIM:613758	6295	**SAG**
OMIM:600059	10594	**PRPF8**	OMIM:250410	10283	**CWC27**
OMIM:276900	10083	**USH1C**	OMIM:268000	23746	**AIPL1**
OMIM:276900	4647	**MYO7A**	OMIM:268000	1259	**CNGA1**
OMIM:180104	6100	**RP9**	OMIM:268000	1406	**CRX**
OMIM:617460	3098	**HK1**	OMIM:268000	6094	**ROM1**
OMIM:614180	7401	**CLRN1**	OMIM:613810	5145	**PDE6A**
OMIM:617304	92840	**REEP6**	OMIM:613660	92211	**CDHR1**
OMIM:617433	23370	**ARHGEF18**	OMIM:612572	3420	**IDH3B**
OMIM:300424	8481	**OFD1**	OMIM:606068	84140	**FAM161A**
OMIM:602772	346007	**EYS**	OMIM:608133	5961	**PRPH2**
OMIM:614181	4117	**MAK**	OMIM:613617	130557	**ZNF513**
OMIM:618345	196	**AHR**	OMIM:607921	25794	**FSCN2**
OMIM:600105	23418	**CRB1**	OMIM:607236	80025	**PANK2**
OMIM:610282	64218	**SEMA4A**	OMIM:616959	51095	**TRNT1**
OMIM:618220	9785	**DHX38**	OMIM:610599	768206	**PRCD**
OMIM:616469	79797	**ZNF408**	ORPHA:436274	2677	**GGCX**
OMIM:613861	79947	**DHDDS**	ORPHA:791	6017	**RLBP1**
OMIM:618195	49855	**SCAPER**	ORPHA:791	8842	**PROM1**
OMIM:601718	24	**ABCA4**	ORPHA:791	7439	**BEST1**
OMIM:613428	388939	**PCARE**	ORPHA:791	54806	**AHI1**
OMIM:600138	26121	**PRPF31**	ORPHA:791	8100	**IFT88**
OMIM:613575	84100	**ARL6**	ORPHA:791	4901	**NRL**
OMIM:615780	55857	**KIZ**	ORPHA:791	55975	**KLHL7**
OMIM:613983	24148	**PRPF6**	ORPHA:791	9129	**PRPF3**
OMIM:613827	2979	**GUCA1B**	ORPHA:791	57670	**KIAA1549**
OMIM:611131	10002	**NR2E3**	ORPHA:791	583	**BBS2**
OMIM:615434	23568	**ARL2BP**	ORPHA:791	145226	**RDH12**
OMIM:610359	23020	**SNRNP200**	ORPHA:791	3419	**IDH3A**
OMIM:617123	55624	**POMGNT1**	ORPHA:791	7275	**TUB**
OMIM:613769	5995	**RGR**	ORPHA:791	762	**CA4**
OMIM:551500	4508	**ATP6**

**Table 2:** List of the 22 HPO terms associated to RP with specificity level between 7 and 12

HPO ID	HPO term	Specificity
HP:0000035	Abnormal testis morphology	9
HP:0000135	Hypogonadism	8
HP:0000405	Conductive hearing impairment	10
HP:0000407	Sensorineural hearing impairment	10
HP:0000431	Wide nasal bridge	8
HP:0000463	Anteverted nares	11
HP:0000518	Cataract	7
HP:0000563	Keratoconus	11
HP:0000602	Ophthalmoplegia	7
HP:0000613	Photophobia	9
HP:0000618	Blindness	8
HP:0000639	Nystagmus	7
HP:0000648	Optic atrophy	9
HP:0000842	Hyperinsulinemia	10
HP:0000987	Atypical scarring of skin	9
HP:0001249	Intellectual disability	7
HP:0001347	Hyperreflexia	7
HP:0005978	Type II diabetes mellitus	9
HP:0007675	Progressive night blindness	7
HP:0007703	Abnormality of retinal pigmentation	8
HP:0008046	Abnormal retinal vascular morphology	11
HP:0008736	Hypoplasia of penis	12

To maintain the specificity and not over-expand the RP mechanistic map, we have selected those genes sharing at least 10 RP-HPO-terms and that were part of signaling pathways according to KEGG DB (Table 3). Finally, the RP mechanistic map was created by merging the circuits related to the core (ORPHA/OMIM) and HPO-amplified sets of RP genes. This resulted in 226 circuits that belong to 40 KEGG signaling pathways (Table 4).

**Table 3:** List of genes sharing ≥ 10 out of the 22 HPO terms related to retinitis pigmentosa for extended RP-mechanistic map

Entrez ID	Gene symbol	Data source	Entrez ID	Gene symbol	Data source
1258	**CNGB1**	ORPHA/OMIM	23568	**ARL2BP**	ORPHA/OMIM
1259	**CNGA1**	ORPHA/OMIM	23020	**SNRNP200**	ORPHA/OMIM
2563	**GABRD**	Amplified by HPO	55624	**POMGNT1**	ORPHA/OMIM
2697	**GJA1**	Amplified by HPO	5995	**RGR**	ORPHA/OMIM
2969	**GTF2I**	Amplified by HPO	26160	**IFT172**	ORPHA/OMIM
3845	**KRAS**	Amplified by HPO	403	**ARL3**	ORPHA/OMIM
3984	**LIMK1**	Amplified by HPO	10461	**MERTK**	ORPHA/OMIM
9569	**GTF2IRD1**	Amplified by HPO	23404	**EXOSC2**	ORPHA/OMIM
51684	**SUFU**	Amplified by HPO	9128	**PRPF4**	ORPHA/OMIM
64218	**SEMA4A**	ORPHA/OMIM	6102	**RP2**	ORPHA/OMIM
4751	**NEK2**	ORPHA/OMIM	50939	**IMPG2**	ORPHA/OMIM
9227	**LRAT**	ORPHA/OMIM	28982	**FLVCR1**	ORPHA/OMIM
5949	**RBP3**	ORPHA/OMIM	5148	**PDE6G**	ORPHA/OMIM
6010	**RHO**	ORPHA/OMIM	6103	**RPGR**	ORPHA/OMIM
9742	**IFT140**	ORPHA/OMIM	123016	**TTC8**	ORPHA/OMIM
7399	**USH2A**	ORPHA/OMIM	55812	**SPATA7**	ORPHA/OMIM
60509	**AGBL5**	ORPHA/OMIM	3614	**IMPDH1**	ORPHA/OMIM
6121	**RPE65**	ORPHA/OMIM	7287	**TULP1**	ORPHA/OMIM
138050	**HGSNAT**	ORPHA/OMIM	10210	**TOPORS**	ORPHA/OMIM
6101	**RP1**	ORPHA/OMIM	6295	**SAG**	ORPHA/OMIM
57709	**SLC7A14**	ORPHA/OMIM	10283	**CWC27**	ORPHA/OMIM
5158	**PDE6B**	ORPHA/OMIM	23746	**AIPL1**	ORPHA/OMIM
26090	**ABHD12**	ORPHA/OMIM	1406	**CRX**	ORPHA/OMIM
112752	**IFT43**	ORPHA/OMIM	6094	**ROM1**	ORPHA/OMIM
375298	**CERKL**	ORPHA/OMIM	5145	**PDE6A**	ORPHA/OMIM
10594	**PRPF8**	ORPHA/OMIM	92211	**CDHR1**	ORPHA/OMIM
10083	**USH1C**	ORPHA/OMIM	3420	**IDH3B**	ORPHA/OMIM
4647	**MYO7A**	ORPHA/OMIM	84140	**FAM161A**	ORPHA/OMIM
6100	**RP9**	ORPHA/OMIM	5961	**PRPH2**	ORPHA/OMIM
3098	**HK1**	ORPHA/OMIM	130557	**ZNF513**	ORPHA/OMIM
7401	**CLRN1**	ORPHA/OMIM	25794	**FSCN2**	ORPHA/OMIM
92840	**REEP6**	ORPHA/OMIM	80025	**PANK2**	ORPHA/OMIM
23370	**ARHGEF18**	ORPHA/OMIM	51095	**TRNT1**	ORPHA/OMIM
8481	**OFD1**	ORPHA/OMIM	768206	**PRCD**	ORPHA/OMIM
346007	**EYS**	ORPHA/OMIM	388939	**PCARE**	ORPHA/OMIM
4117	**MAK**	ORPHA/OMIM	6017	**RLBP1**	ORPHA/OMIM
196	**AHR**	ORPHA/OMIM	8842	**PROM1**	ORPHA/OMIM
23418	**CRB1**	ORPHA/OMIM	7439	**BEST1**	ORPHA/OMIM
9785	**DHX38**	ORPHA/OMIM	54806	**AHI1**	ORPHA/OMIM
79797	**ZNF408**	ORPHA/OMIM	8100	**IFT88**	ORPHA/OMIM
79947	**DHDDS**	ORPHA/OMIM	4901	**NRL**	ORPHA/OMIM
49855	**SCAPER**	ORPHA/OMIM	55975	**KLHL7**	ORPHA/OMIM
24	**ABCA4**	ORPHA/OMIM	9129	**PRPF3**	ORPHA/OMIM
2677	**GGCX**	ORPHA/OMIM	57670	**KIAA1549**	ORPHA/OMIM
26121	**PRPF31**	ORPHA/OMIM	583	**BBS2**	ORPHA/OMIM
84100	**ARL6**	ORPHA/OMIM	145226	**RDH12**	ORPHA/OMIM
55857	**KIZ**	ORPHA/OMIM	3419	**IDH3A**	ORPHA/OMIM
24148	**PRPF6**	ORPHA/OMIM	7275	**TUB**	ORPHA/OMIM
2979	**GUCA1B**	ORPHA/OMIM	762	**CA4**	ORPHA/OMIM
10002	**NR2E3**	ORPHA/OMIM	4508	**ATP6**	ORPHA/OMIM

Source specifies whether the gene was already on the ORPHA/OMIM RP core set or it is HPO amplified

**Table 4:** KEGG pathways involved in the Retinitis Pigmentosa mechanistic map with mapped the RP-genes

KEGG pathway name	KEGG-ID	RP genes mapped
MAPK signaling pathway	hsa04010	KRAS
ErbB signaling pathway	hsa04012	KRAS
Ras signaling pathway	hsa04014	KRAS
Rap1 signaling pathway	hsa04015	KRAS
cGMP-PKG signaling pathway	hsa04022	CNGB1, CNGA1, GTF2I, GTF2IRD1
cAMP signaling pathway	hsa04024	CNGB1, CNGA1
Chemokine signaling pathway	hsa04062	KRAS
HIF-1 signaling pathway	hsa04066	HK1
FoxO signaling pathway	hsa04068	KRAS
Sphingolipid signaling pathway	hsa04071	KRAS
Phospholipase D signaling pathway	hsa04072	KRAS
mTOR signaling pathway	hsa04150	KRAS
PI3K-Akt signaling pathway	hsa04151	KRAS
Apoptosis	hsa04210	KRAS
Longevity regulating pathway—mammal	hsa04211	KRAS
Hedgehog signaling pathway	hsa04340	SUFU
Axon guidance	hsa04360	SEMA4A, KRAS, LIMK1
VEGF signaling pathway	hsa04370	KRAS
Tight junction	hsa04530	KRAS
Gap junction	hsa04540	GJA1, KRAS
Signaling pathways regulating pluripotency of stem cells	hsa04550	KRAS
Natural killer cell mediated cytotoxicity	hsa04650	KRAS
T cell receptor signaling pathway	hsa04660	KRAS
B cell receptor signaling pathway	hsa04662	KRAS
Fc epsilon RI signaling pathway	hsa04664	KRAS
Fc gamma R-mediated phagocytosis	hsa04666	LIMK1
Neurotrophin signaling pathway	hsa04722	KRAS
Cholinergic synapse	hsa04725	KRAS
Serotonergic synapse	hsa04726	KRAS
GABAergic synapse	hsa04727	GABRD
Regulation of actin cytoskeleton	hsa04810	KRAS, LIMK1
Insulin signaling pathway	hsa04910	HK1, KRAS
GnRH signaling pathway	hsa04912	KRAS
Progesterone-mediated oocyte maturation	hsa04914	KRAS
Estrogen signaling pathway	hsa04915	KRAS
Melanogenesis	hsa04916	KRAS
Prolactin signaling pathway	hsa04917	KRAS
Thyroid hormone signaling pathway	hsa04919	KRAS
Oxytocin signaling pathway	hsa04921	KRAS
Aldosterone-regulated sodium reabsorption	hsa04960	KRAS

*RP* retinitis pigmentosa

To functionally annotate the RP Mechanistic Map, we used UniprotKB annotations and the GO ontologies. From all the annotations obtained for each circuit, based on RP biological mechanisms described in the literature, we prioritized those functions related to RP progression by thorough manual curation. The GO/Uniprot functions as well as the hallmarks annotating each circuit of the RP Map are shown in Additional file 2: Table S2.

Filtered functional annotations of the RP Map converged into 9 RP related processes that we defined as the RP hallmarks: “Apoptosis”, “Necrosis”, “Stress Response”, “Inflammatory response”, “DNA integrity”, “Fatty acids and lipid metabolism”, “Sensory and stimuli response”, “Development processes” and “Neuronal processes”. A summary of the percentage of hallmark modules by pathways is depicted in Additional file 11: Fig. S1B. Pathways with a higher number of circuits, like cyclic adenosine monophosphate (cAMP) dependent signaling pathway (hsa04024), forkhead box O (FoxO) signaling pathway (hsa04068), Ras signaling pathway (hsa04014), and PI3K-Akt signaling pathway (hsa04151) show a homogeneous high coverage over the hallmarks modules. In contrast, pathways with fewer circuits (smaller in the RP map), like GnRH signaling pathway or VEGF signaling pathway, show more specificity towards specific hallmark modules like “Apoptosis” and “Development processes” (GnRH signaling pathway) or “Fatty acid and lipid metabolism” and “Inflammatory response” (VEGF signaling pathway). Likewise, the distribution of the hallmarks among the pathways is highly dependent on how broad the hallmark is. Broader hallmarks are related to many pathways compared to hallmarks that tag fewer circuits and hence fewer pathways. A summary of the percentage of hallmark modules by pathways, as well as the percentage of coverage of each hallmark on the RP mechanistic Map, are depicted in Additional file 11: Fig. S1B, C and the percentage of coverage of each hallmark on the RP mechanistic Map is depicted in Additional file 11: Fig. S1C.

The information regarding RP mechanistic map, pathways, and circuits topology is openly available at the Hipathia RP viewer, and accessible at http://hipathia.babelomics.org/RP_Mechanistic_Map/ [35].

**Fig. 2:**
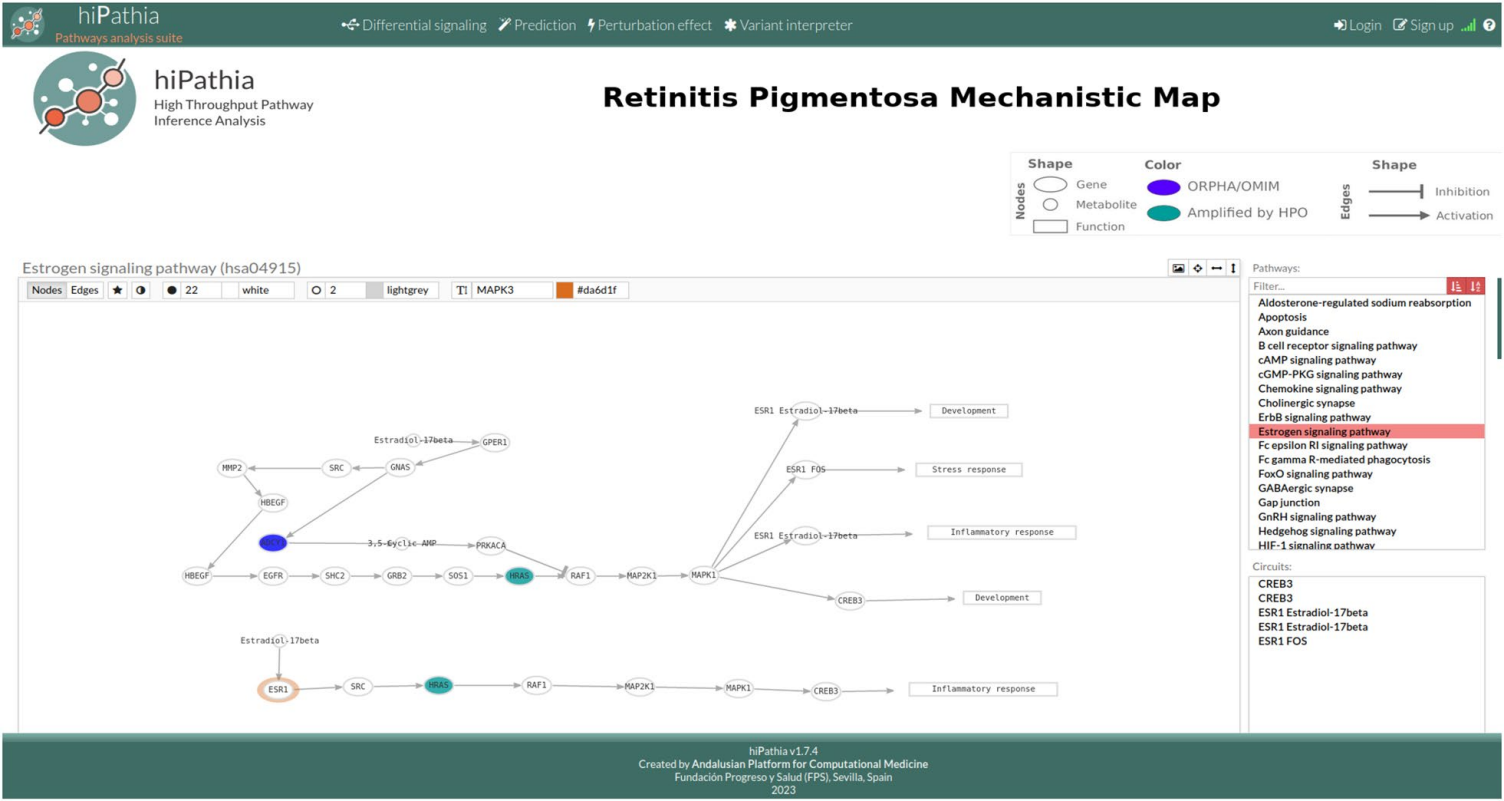
Overview of the RP mechanistic map viewer in the Hipathia Web tool. The RP mechanistic map is composed of 226 circuits belonging to 40 pathways. The Estrogen signaling pathway is shown as a selection example. Colors represent RP genes found to be associated with the disease in OMIM/ORPHANET (Blue) or to be related to the disease phenotypes in HPO db (green). The shape of the edge indicates the type of interaction (activation/inhibition) between circuit nodes. The left tab above the main panel enables the selection/search of specific proteins on the selected pathway, while the right tab allows the option to export as an SVG image the visualized pathway

The Hipathia RP viewer allows the user to explore the elements and filter the circuits or pathways desired. It shows the interaction among the protein nodes, the ORPHA/OMIM, and HPO amplified genes in different colors, as well as the RP-hallmark related to each circuit. The user can search for specific genes based on their gene symbol or entrez ID, or for specific hallmarks of RP from the 9 described. Figure 2 illustrates the overall layout of the Hipathia RP viewer and the RP Map.

#### Approved drug targets selection

To assess the potential effect of actionable therapeutic targets over the RP Mechanistic Map we have used DrugBank 5.1.8 DB. From 5004 targets, 7919 drugs, and a total of 26,979 KDT-drug combinations parsed, we have included 711 KDTs from 1410 approved drugs with known pharmacological action and measurement values in the GTEx V8 dataset. In total, we included 2688 combinations of drug-KDT that can be found in Additional file 3: Table S3. The drug-KDT effects were simplified into 5 types resulting in 841 drug-KDT “activators”, 1530 drug-KDT “inhibitors”, 24 drug-KDT modulators, 192 drug-KDT “ligands”, and 101 drug-KDT with various effects categorized into “other”.

#### Machine learning model performance

**Fig. 3:**
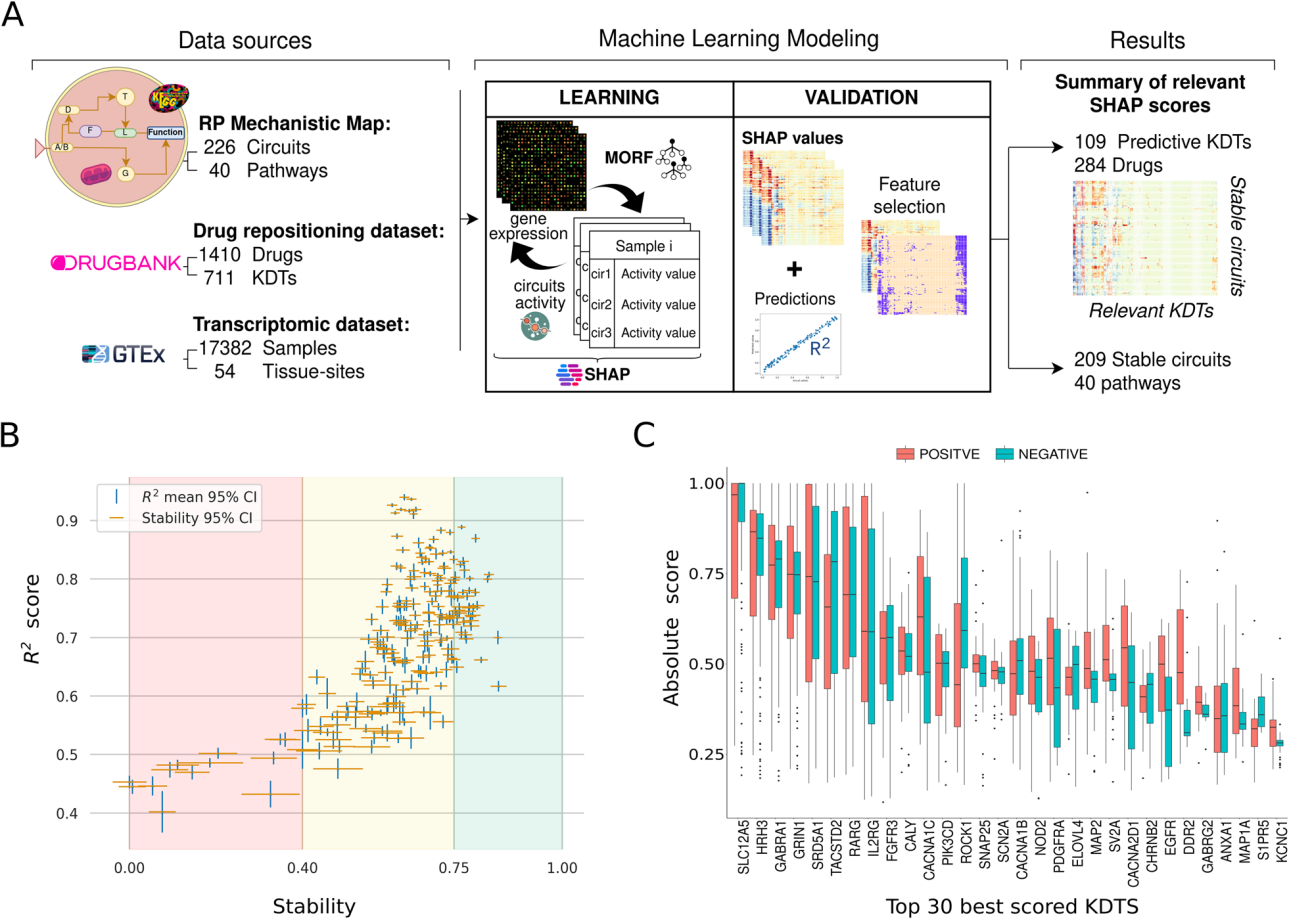
Retinitis pigmentosa mechanistic model from the inside. **A** Overview of the Retinitis Pigmentosa machine learning-driven approach to drug repurposing. A Multi-output random Forest (MORF) regressor was trained, with gene expression from the GTEx dataset, and the circuit’s activity values, to assess the predictive power of 711 KDTs over the activity of the 226 circuits that configure the RP mechanistic map. Feature selection of highly predictive KDTs for a given circuit was calculated based on the R2 score and up to the top 5% KDTs ranked by the mean absolute Shapley value. **B** Machine learning model (MORF) stability vs R2 score circuit metrics. **C** Boxplots representing the distribution of the absolute model relevances (Y-axis) across the top 30 ranked KDT (X-axis). The rank is obtained by means of the L1 norm and the values are colored by their sign (positive = red; negative = blue)

Altogether, applying the proposed drug repositioning methodology to RP, we have identified 109 KDTs, belonging to 284 drugs (Fig. 3A), whose expression patterns could potentially modulate the activity of the RP mechanistic map circuits. All the KDTs × RP circuits model scores can be found in Additional file 4: Table S4. The selection of relevant KDTs is depicted in a boolean matrix in Additional file 5: Table S5. Results from the model performance are shown in Fig. 3B and Additional file 6: Table S6 as the 95% confidence intervals for the mean R^2^ and the Nogueira stability estimate [40] for each specific circuit. Since the trustability of the results relies on stability across the ML model predictions, 207 circuits with a stability score above 0.4 (the empirical lower effect size threshold proposed in [40]) were filtered from the 226 RP mechanistic map circuits.

#### Relevance of known-drug target genes in retinitis pigmentosa mechanistic map

The model selected 109 KDTs out of 207 circuits that compose the RP map and assigned a relevance score to each KDT-circuit pair (see “Methods”). The score shows the KDT’s impact on the circuit, either positively or negatively. The rescaled score (0–1) distribution of the top 30 scoring KDTs is depicted in Fig. 3C, highlighting the positive/negative regulating role of certain KDTs which could potentially indicate which drugs, based on their effect over the KDT, would better revert the disease effects.

**Fig. 4:**
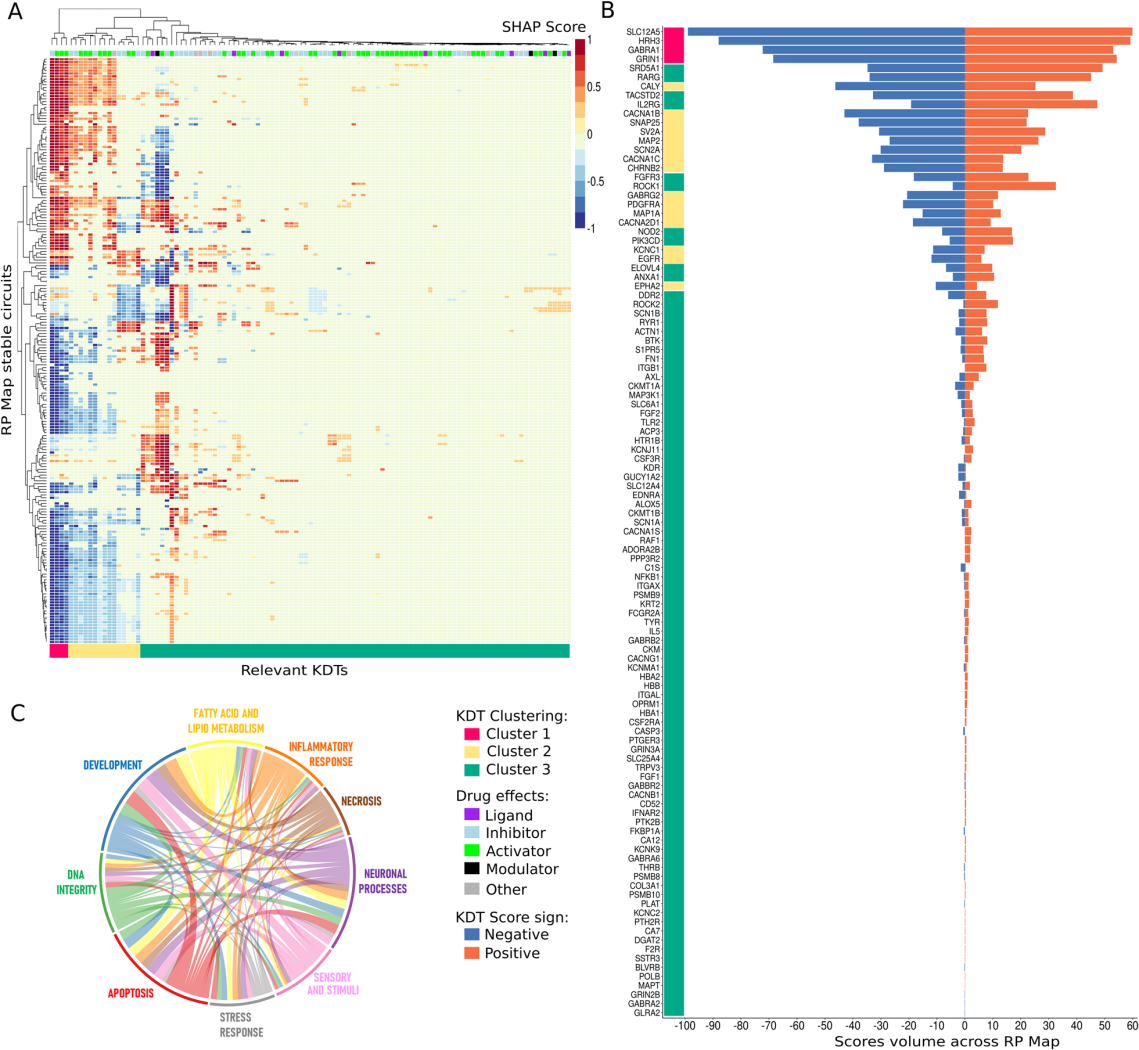
Influence and profile of KDTs on Retinitis Pigmentosa mechanistic map. **A** Heatmap of the 109 relevant predictive KDTs (X-axis) normalized (− 1, 1) SHAP scores over the stable circuits of the RP Map (Y-axis). The sign indicates the direction of the KDT influence over each specific circuit and the score value depicts how strong is the influence of a specific KDT for predicting the activity of a specific circuit. The top color bar represents the most frequent drug effects of that specific target (KDT). At the bottom, the KDT groups obtained by hierarchical clustering are represented by red (cluster 1 with 4 KDTs), yellow (Cluster 2 with 15 KDTs), and green (Cluster 3 with 90 KDTs) color. B) Barplot representing the distribution of SHAP scores showing the accumulated volume of positive (in red) and negative (in blue) scores (X-axis), for each specific KDT (Y-axis) overall RP Map. On the left side, KDTs are annotated with their gene symbol and colored by their cluster. **C** Chord diagram representing the shared influence of relevant KDTs over the circuits belonging to the 9 RP hallmarks modules. The size of the chord joining two hallmarks represents the number of shared KDTs, with their SHAP scores weighted by the % of the hallmark covered (no. of circuits tagged by the hallmark with the KDT/total number of circuits with the hallmark)

To exploit the full potential of the results, we generated an annotated heatmap with the relevant KDTs as columns, the stable circuits as rows, and annotations on the most frequent drug effect over each relevant KDT on top (Fig. 4A). The intensity of the color represents the strength of the KDT interaction over a specific circuit, while the sign of the interaction is shown in blue (negative) or red (positive). Furthermore, to gain insight into the KDTs scores and their structure, we used hierarchical clustering of the resulting relevance matrix and plotted the distribution of scores of each relevant KDT (Fig. 4C). We used the Gap statistic among other metrics to determine the optimal number of clusters (Additional file 12: Fig. S2A), resulting in a total of 3 clusters, cluster 1 represented by 4 KDTs, cluster 2 by 15 KDTs and cluster 3 by 90 KDTs (Additional file 7: Table S7). As we can observe in Fig. 4A, a small group of KDTs, from cluster 1, are strongly influencing the activity of most circuits. Interestingly, these 4 KDTS, are either part of GABA and glutamate receptors (GABRA1 and GRIN1), related to important neuronal-development processes, involved in chemical synaptic transmission (HRH3), or involved in ion transmembrane transport processes (SLC12A5). These processes, which play important roles in various aspects of retinal function, were also highlighted by the results obtained in the Gene Ontology (GO) enrichment analysis (Additional file 12: Fig. S2C) of cluster 1. On the other side, KDTs belonging to clusters 2 and 3 seem to influence only certain regions of the RP mechanistic Map, showing a higher specificity. Results from the GO enrichment analysis show that cluster 2 is strongly focused on ion transport and synaptic signaling processes while cluster 3, which exhibits a high variability (Additional file 12: Fig. S2B), embraces a wide range of biological processes, some of them related to tissue remodeling and cell migration. Additionally, cluster 3 is associated with processes related to cellular response to hormones and immune response, while cluster 2 is more focused on nervous system development and cellular communication. Despite the differences, all clusters share some commonalities such as processes related to the regulation of ion transport as well as to cellular response to chemical stimulus, regulation of cellular component organization, and the activity of neuronal receptors (Additional file 7: Table S7, Additional file 12: Fig. S2C).

Furthermore, the distribution of KDT scores depicted in Fig. 4B highlights the dual role (positive = activating; negative = repressing) that proteins might exhibit depending on the cellular process they are involved in. Although most KDTs show a balance between positive and negative scores, others like FGF2 and ROCK1 seem to have a higher proportion of positive scores condensed in fewer circuits, which are mostly related to Development and Neuronal Hallmarks. Due to the proportions of the results, only clusters are shown in the Heatmap. An explorable version of the Heatmap with circuit names and KDT symbols can be accessed at Additional file 13: Fig. S3.

In order to assess how the RP hallmarks were being modulated by the KDTs, we identified the shared KDTs among the circuits belonging to each of the RP hallmark modules, giving weights to the interaction based on the KDT mean score. As observed in Fig. 4C, there is a strong link between the “Neuronal processes”, “Development”, and “Apoptosis” hallmark modules. Overall, since one circuit can be tagged by multiple hallmarks when aggregating the influence of KDTs, most hallmarks are influenced by a wide range of KDTs.

In our study, we utilized a functional profiling approach to select KDTs for validation in *rd10* mice. By gathering the set of annotations linked to the RP map circuits into the 9 RP hallmarks, we can (functionally profile the KDTs) assess how a relevant KDT affects each functional module defined by the hallmarks. Our Disease Map, linked to nine RP-associated hallmarks, prioritized KDTs mostly affecting circuits central to neuronal and development processes. Taking into account that Retinitis Pigmentosa (RP) progression encompasses not only photoreceptor cell dysfunction but also remodeling of the inner retina and its synaptic connections, our emphasis was on neurotransmitter-mediated communication. Key to this communication are glutamate and GABA, governing vertical and lateral retinal neuron communications, respectively. Given their significance in maintaining visual system integrity and their roles in neuronal and developmental processes, we specifically selected top-scored KDTs associated with receptors of glutamatergic, GABAergic, and Glycinergic neurotransmission. These KDTs were chosen based on their prominent functional roles in these RP-related processes, providing a focused approach to our experimental validation.

### Dysregulation of GABA, glycine, and glutamate receptor subunits in *rd10* mice

**Fig. 5:**
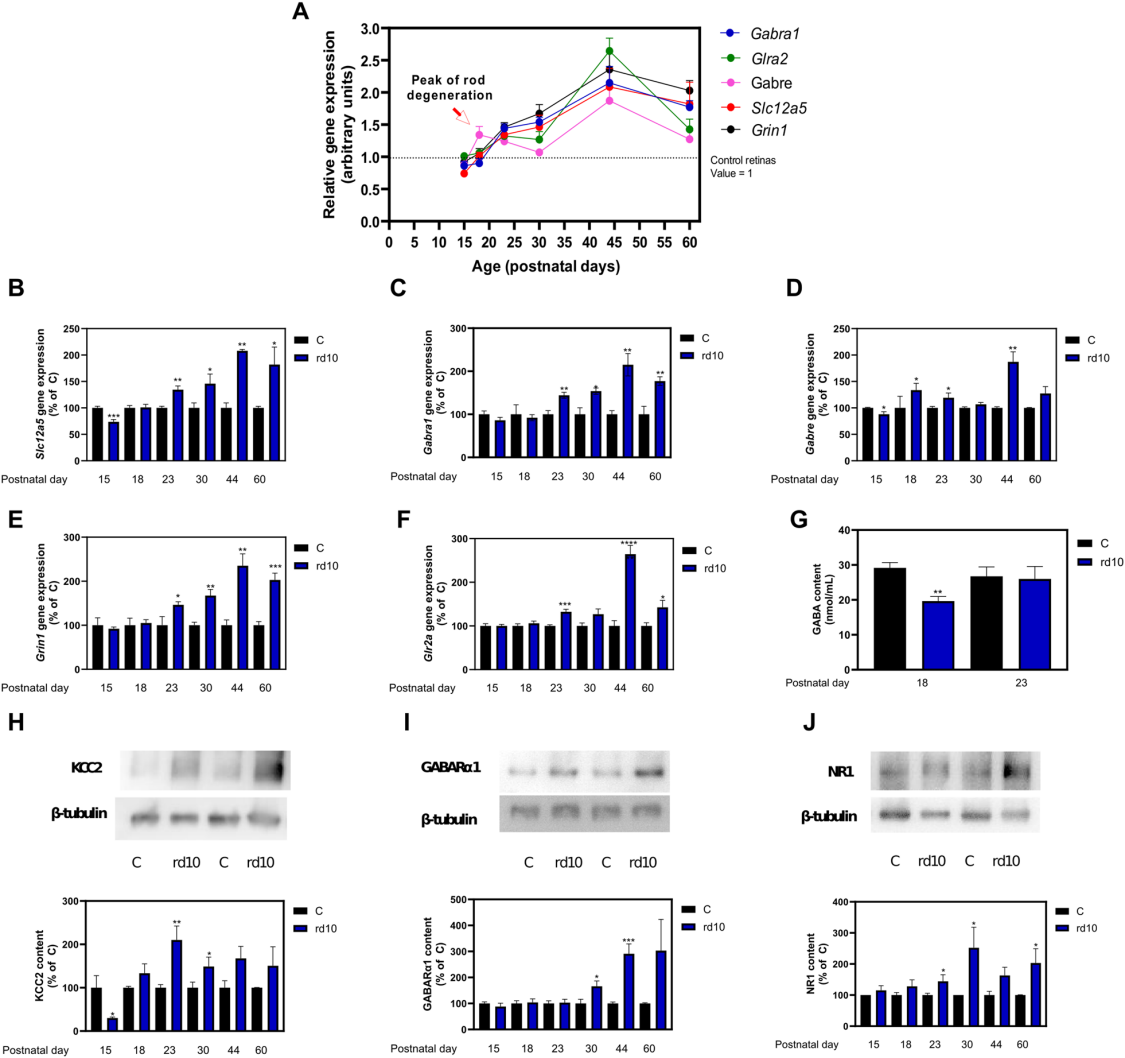
Experimental validation of selected KDTs in *rd10* mice, murine model of Retinitis Pigmentosa. Normalized gene expression profiles of selected KDTs from postnatal day (P) 15–60 with the observed peak of rod degeneration at P18 (**A**). Gene expression of K+/Cl− co-transporter 2 (*Slc12a5*) (**B**), alpha 1 and epsilon subunits of GABA A receptor (*Gabra1 and Gabre*) (**C**, **D**), glutamate ionotropic receptor NMDA type subunit 1 (*Grin1*) (**E**), and glycine receptor alpha 2 subunit (*Glra2*) (**F**), retinal GABA concentration in retinal extracts (**G**, the protein content of KCC2 (**H**) and GABA_R_α1 (**I**) and NR1 (**J**) with representative western blots at P23 in control and *rd10* mice. The unpaired t-test was used to compare control “C” and *rd10* mice. *p < 0.05; **p < 0.01; ***p < 0.001. All data were presented as the mean ± standard error of the mean (SEM). We analyzed at least eight retinas for each group and age

**Fig. 6:**
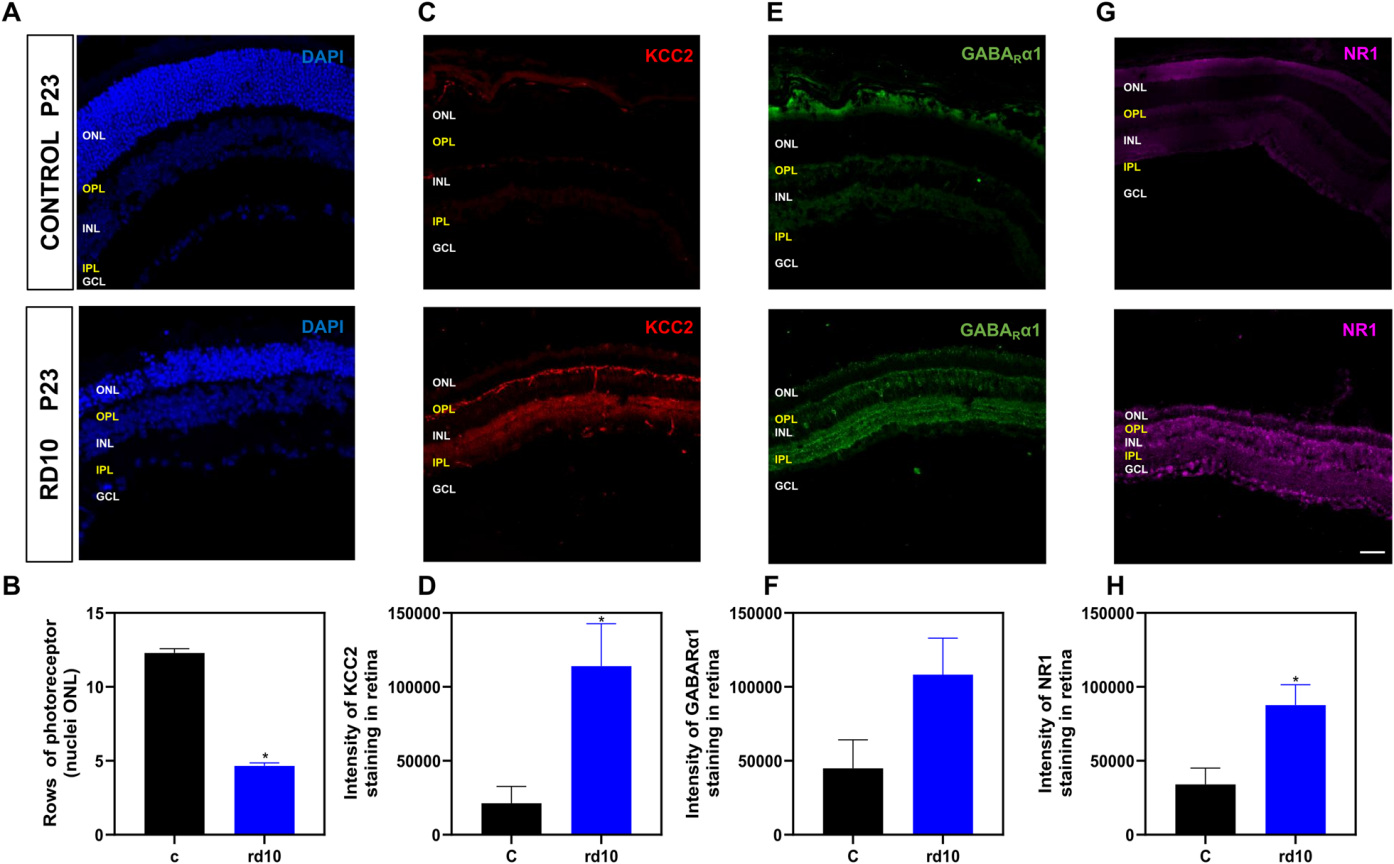
Histological study of selected targets in retinas sections of *rd10* mice, murine model of Retinitis Pigmentosa. Confocal micrographs of transverse sections through *rd10* mice retina. DAPI nuclear staining illustrating the retinal cytoarchitecture (retinal inner nuclear layer INL, retinal inner plexiform layer IPL, retinal outer nuclear layer ONL, retinal outer plexiform layer OPL and retinal ganglion cell layer *GCL*). Photoreceptor degeneration (**A**, **B**), and retinal distribution of selected KDTs KCC2 (**C**, **D**), GABA_R_α1 (**E**, **F**) and NR1 (**G**, **H**) in control and *rd10* mice at P23. The Mann–Whitney test was used to compare control “C” and *rd10* mice. Scale bar: 50 µm. *p < 0.05. All data were presented as the mean ± standard error of the mean (SEM). We analyzed at least four retinas for each group and age

In this study, we evaluated the overall impact of target genes for the GABA A receptor subunits alpha and epsilon (*Gabra1* and *Gabre*), the glutamate ionotropic receptor NMDA type subunit 1 (*Grin1*), the glycine receptor alpha 2 subunit (*Glra2*), the solute carrier family 12 member 5 (*Slc12a5) that* encodes K+–Cl− cotransporter 2 (KCC2) in *rd10* mouse retinas compared to age-matched control retinas from postnatal days P15 to P60 (Fig. 5). In addition, we previously evaluated the role of target genes lipoxygenase 5 (*Alox5*) and elongase 4 (*Elovl4*), both related to fatty acid metabolism, in the mechanisms of RP at early postnatal days [48]. We observed a significant downregulation of *Slc12a5* gene (Fig. 5A, B) and a less acute downregulation of *Gabra1* and *Gabre* genes at P15, just before the peak of rod degeneration happens to be observed (P18) (Fig. 5A, C, D). At P18, we detected an upregulation for *Gabre* gene accompanied by a decrease in GABA content in retinal homogenates (Fig. 5A, D, E). We detected significant upregulation of *Slc12a5*, *Gabra1, and Gabre* genes from P23 to P60 (Fig. 5A–D). The upregulation in gene expression of *Slc12a5 and Gabra1* was confirmed by a western blot of their proteins KCC2 and GABARα1 (Fig. 5H, I). As shown in Fig. 6A, photoreceptor degeneration is quite evident at P23. At this age the number of nuclei remaining in the outer nuclear layer (ONL) in *rd10* retinas was significantly lower than in age-matched control retinas. We also performed an immunostaining showing their localization mainly at the OPL, INL and IPL at P23 (Fig. 6B, C). Retinas from *rd10* mice showed a higher content of KCC2 and GABARα1 than age-matched retinas from control mice at the inner retina at this age (Fig. 6B, C). At P18 we did not observe significant changes in the target genes *Glra2* or *Grin1*. However, from P23 to P60 both genes were upregulated (Fig. 5A E, F. The upregulation in gene expression of *Grin1* was also confirmed by western blot and immunostaining of its protein NR1 (Figs. 5J, 6D). We previously described that *Elovl4* gene was dramatically decreased in retinas from *rd10* mice compared to age-matched retinas from control mice from P13 to P60 (data not shown). Besides, we showed downregulation of *Alox5* gene in the early stages of RP, up to P23, and from there it started to gradually increase until it was significantly upregulated at P60 (data not shown). Taking together these findings, we proposed that RP leads to an altered synthesis of specialized pro-resolving mediators which are involved in the resolution of inflammation [44] (data not shown). All data can be accessed in Additional file 8: Table S8.

### Functional profiling of drugs targeting candidate genes

**Fig. 7:**
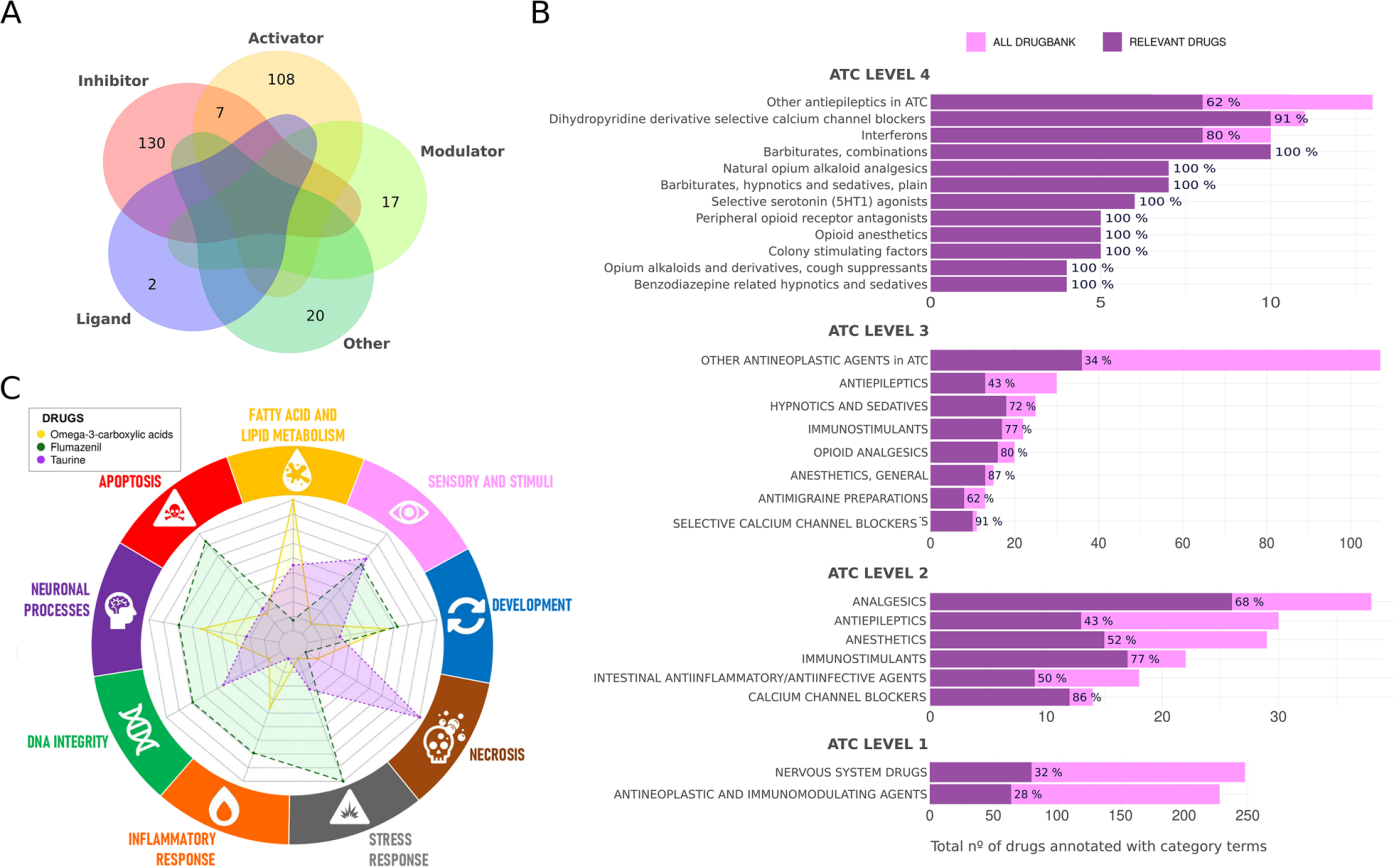
Functional profile of potentially repurposable drugs. **A** Venn diagram representing the simplified pharmacological actions of drugs, collected in Drugbank 5.1.8 db, with at least one target (KDT) predicted as relevant by our model. **B** Over-representation analysis of drugs with relevant KDTs over the ATC categories (Y-axis) across the 4 ATC levels, showing the total number of drugs that fall into each category (X-axis) and the percentage of coverage of relevant drugs over the parsed DrugBank 5.1.8 db. Only ATC categories with an FDR p-value ≤ 0.05 are shown. Fisher’s exact test was used to identify overrepresented ATC categories in our relevant results compared to their proportions in the parsed DrugBank 5.1.8 db. **C** Spider plot illustrating the profile of selected promising drug candidates, Omega-3 carboxylic acid, Flumazenil, and Taurine, targeting experimentally validated targets ELOVL4, GABRA1 and GLRA2 respectively, over Retinitis Pigmentosa functional hallmarks. Drug candidates were selected based on having a pharmacological action with the potential to revert the observed RP effects in the expression patterns of their targets (relevant KDTs), measured on *rd10* RP murine models

Out of 1410 drugs, 284 were found to target relevant predicted KDTs. Details of these drug-KDT interactions can be found in Additional file 9: Table S9. The distribution of drug effects is shown in Fig. 7A. Over-represented categories from the different ATC levels with an FDR-adjusted p.value < 0.5 are shown in Fig. 7B and provided in Additional file 10: Table S10. The ATC classification ranges from broad categories (ATC level 1) to specific ones (ATC level 4), excluding the compound names (ATC level 5). Figure 7B reveals an enrichment in Nervous-system and Antineoplastic-Immunomodulating drugs at ATC level 1. Deeper levels highlight a significant presence of calcium channel blocker drugs, with 86% coverage at ATC level 2 and 91% at level 3. ATC level 4 also indicates a high concentration of interferon drugs, serotonin agonists, barbiturates, and various analgesic drugs.

Based on experimentally validated KDTs, we identified drugs, listed in Table 5, whose effects over their targets could potentially reverse the disease effects (the UP/DOWN regulation observed in *rd10* mice). These include drugs for GABA receptors, ELOVL4, and Glycine receptors. To understand the full drug impact on the RP Mechanistic Map, we measured how the drugs would influence the RP hallmark modules, based on the scores we obtained for their targets. Figure 7C shows that compounds like Omega-3 and fish oils significantly affect the “Neuronal”, “Inflammatory response”, and “Fatty Acids and Lipid processes” modules. Flumazenil, a GABA receptor antagonist, primarily impacts the “Stress Response” module. Taurine, vital for many biological functions, notably affects the “Necrosis” and “Sensori and stimuli” modules, aligning with its cytoprotective properties and high presence in excitatory tissues.

**Table 5:** Drugs targeting validated KDTs with the potential to revert RP effects, highlighting functionally studied promising drug repositioning candidates

Gene	Drug name	DrugBank ID	Pharmacological action
ALOX5 (240)	Sulfasalazine	DB00795	Inhibitor
ALOX5 (240)	Diethylcarbamazine	DB00711	Inhibitor
ALOX5 (240)	Mesalazine	DB00244	Inhibitor
ALOX5 (240)	Balsalazide	DB01014	Inhibitor
ALOX5 (240)	Masoprocol^a^	DB00179	Inhibitor
ALOX5 (240)	Meclofenamic acid	DB00939	Inhibitor
ELOVL4 (6785)	Omega-3-carboxylic acids	DB09568	Potentiator
GABRA1 (2554)	Methohexital	DB00474	Antagonist
GABRA1 (2554)	Flumazenil	DB01205	Antagonist
GLRA2 (2742)	Taurine	DB01956	Agonist
GRIN1 (2902)	Orphenadrine	DB01173	Antagonist
SLC12A5 (57468)	Bumetanide	DB00887	Inhibitor

^a^Masoprocol was withdrawn from the U.S. market in June 1996

## Discussion

In this work, we propose a unique approach that, starting from the set of disease-affected genes, provides a comprehensive landscape of the molecular mechanisms of the RP disease along with its druggable space. The method establishes the RP mechanistic disease map as an actionable environment and employs an explainable machine-learning model to assess the influence of druggable molecules, like KDTs, over the disease environment. Our approach merges information from transcriptomics, pathway graphs, biological/clinical DBs, and drug-target interactions, to generate an in-depth view of the disease. The novelty of this workflow lies in the integration of multiple data sources, reinforcing interpretability with biological knowledge while reducing the dimensionality of the datasets.

Our methodology has enabled us to create a comprehensive yet straightforward map of RP, representing its functional landscape. The utility of this method relies on its interpretability. Artificial intelligence is often used in repurposing methods to compensate for the lack of prior knowledge. However, rational target discovery and drug repositioning can be a useful addition when prior knowledge is available, as it can help fill in the gaps and limit the unknown variables. By employing mechanistic models grounded in solid biological knowledge, we have managed to reduce dimensionality by providing a causal link between gene expression and functional cell behavior, and assess the impact of specific targets on different parts of the mechanistic map. This is particularly beneficial for diseases with diverse genotypic presentations and complex pathophysiology, such as RP [5].

Retinitis pigmentosa (RP) is a heterogeneous genetic disorder that leads to the progressive degeneration of photoreceptor cells in the retina, eventually leading to blindness. Although the pathogenesis of RP is complex, as part of this work, we have identified key features of the disease. Based on the signaling circuits identified by our methodology, and supported by the scientific literature, we have defined nine hallmarks of RP: “Apoptosis”, “Necrosis”, “Oxidative stress”, “Inflammatory response”, “DNA integrity”, “Neuronal processes”, “Fatty-acid–lipid metabolism”, and “Sensory and stimuli response”.

Photoreceptor cells, like other neuronal cells, have a postmitotic nature that allows for extended cell life beyond normal physiological conditions, but it may also induce abnormal photoreceptor cell death [45, 46]. We have indeed defined cell death (“Apoptosis” and “Necrosis”) as RP hallmarks within the proposed RP actionable map.

In RP, apoptosis is the major mechanism of photoreceptor cell death, involving the activation of caspases or certain members of the TNF receptor family [47], triggered both by intracellular stress signals, such as DNA damage or oxidative stress, processes defined as RP hallmarks, or by the binding of extracellular ligands to death receptors on the cell surface [43, 48].

Necrosis also has been linked to retinal degeneration as the main mechanism responsible for massive uncontrolled photoreceptor cell death [49], necrosis-like processes can happen rapidly and are linked to a failure in bioenergetics [46]. Interestingly, receptors that initiate cell death are part of the TNF superfamily, a family of cytokines involved in inflammation (another defined hallmark of RP) and in several pathways within our defined RP map [50, 51]. Inflammation is usually mediated by TNFα, IL-6, IL-1α, and IL-1β, among other mediators, all of which are present in RP patients and murine models of the disease [52–54], regulating the production of chemokines and cytokines [55–58]. Moreover, TNFα can promote, via RIPK3-MLKL signaling, the activation of NLRP3 inflammasome, a major multiprotein that induces inflammatory-mediated immune cell infiltration, accelerating necrosis-like photoreceptor cell death [58–60].

Oxidative stress is another critical component of the pathogenesis of RP that is present in both the RP map and the predicted relevant KDTs. In RP, oxidative stress is initiated by the release of ROS from dying photoreceptor cells and microglia cells. The accumulation of ROS leads to the activation of additional stress pathways, including the unfolded protein, leading to cell death [44, 60–62].

We also observed the implication of energetic and glucose-related functions, including HIF1 and HK1 pathways. Both HKI and HKII isoforms are expressed in the retina and target genes of the HIF-1 pathway, part of the RP map [58]. In fact, several cases of mutations in the HK1 gene were reported to cause RP in humans [4, 5]. This could affect glucose metabolism and energy supply to retinal cells, given that photoreceptor cells have very high energetic requirements.

Since neurons are highly excitable and sensitive to ischemia-induced death, due to their high ATP turnover rate [48, 63], the presence of voltage-gated sodium or calcium channels makes neurons susceptible to overload and swelling, while glutamate-gated channels make neurons susceptible to excitotoxicity and rupture [64–66]. Thus, stimuli and sensory transduction also plays a role in RP progression, making photoreceptor cells and other neural cells susceptible to excitotoxic neurotransmitters, such as glutamate, or to the action of other neurotransmitters, like GABA or glycine [67]. As mentioned above, RP leads to a progressive loss of photoreceptor cells, but other neural cells suffer morphologic and metabolic changes resulting in retinal remodeling including loss of retinal ganglion cells (RGCs) or network rewiring [68].

As commented above, gene defects primarily lead to the dysfunction and death of photoreceptor cells and many therapeutic approaches are focused on these cell types (outer retina). However, as RP progresses a remodeling of the inner retina also occurs including formation of abnormal neural circuits and changes in the electrophysiologic properties of the retinal network [69–71]. After photoreceptor cell death, the inner retina changes their synaptic connections affecting the transmission of information [72]. The communication between retinal neurons is mediated electrical but also chemical signals, the former includes the neurotransmitters glutamate, and GABA and their receptors. Glutamate mediates vertical communication between photoreceptor-bipolar cells and bipolar-ganglion cells. While GABA mediates lateral communication via horizontal and amacrine cells. The maintenance of the integrity of the visual systems beyond photoreceptors is a limitation for the successful outcome of any retinal treatment. In this study, we selected as KDT some subunits of the receptors associated to the glutamatergic, GABAergic and Glycinergic neurotransmission because changes in these receptors would have a significant impact in retinal cross-talk. In addition, the understanding of the time-course of the retinal remodeling may be important for achieving a proper window of therapeutic intervention [69].

In this study, we predicted and experimentally validated that RP is accompanied by alterations in neurotransmitter subunits, such as glutamate, GABA, and glycine. In many types of horizontal and amacrine cells, GABA serves as the main inhibitory neurotransmitter, in addition to glycine, which is known to also have an inhibitory role [73]. Retinal activity functions required a balance between excitation (glutamate) and inhibition (GABA and glycine). By increases in chloride conductance, GABA causes membrane hyperpolarization. Active chloride extrusion is achieved via the coupled transport of potassium and chloride ions via K–Cl cotransporters (KCC), with KCC2 being the primary active chloride extrusion system responsible for GABAergic systems. Hence, the changes in KCC2 expression (predicted and validated) could affect the GABAergic driving force [74]. Indeed, the ionotropic GABA A receptors (consisting of five subunits) are predicted as relevant KDTs, and found in almost all retinal cells [75, 76]. Inhibitory neurons seem to influence how the excitatory neurons integrate information through synaptic interactions. Under physiological conditions, retinal inhibition modulates the threshold responses of RGCs under ambient light, selectively masking certain signals to ensure the appropriate ones cross the optic nerve. GABA A receptor seems to be involved in the dynamic control of this masking inhibition and serves as a mechanism for neuronal adaptation. The upregulation of the alpha 1 and epsilon subunits of the GABA A receptor (Fig. 5C, D) observed in *rd10* retinas could potentially change the threshold of RGCs activities leading to modifications in the perceived visual environment [77].

RP leads to photoreceptor degeneration triggering changes in the retinal morphology in human and animal models of RP. Studies on *rd1* mice, carrying a mutation on the exon 7 of the *Pde6b* gene encoding the beta subunit of *cGMP-PDE*, with a greater rate of degeneration than *rd10* mice, show that cone-mediated GABAergic amacrine cells displayed functionally altered NMDA receptors (such as *Grin1*) and high levels of GABA in rod bipolar cells, processes predicted by our model and validated as significantly affected in *rd10* mice (Figs. 5E–J, 6 and Additional file 14: Fig. S4). In other studies, the use of GABA receptor antagonists in P23H rats (RP animal model carrying a missense mutation in the *rhodopsin (rho)* gene) reduces the observed stimulation thresholds of RGCs [78, 79]. Interestingly, the use of picrotoxin, an antagonist for GABA A/C and glycine receptors, also results in the blockade of the inhibitory mechanisms and ameliorates visual function in *rd10* mice [80].

Although the role of GABAergic systems in RP is not completely established, our data suggest that the GABAergic system may be overactive during RP progression. We observed a significant increase in *Gabra1* and *Slc12a5* genes expression, their respective proteins GABARα1 and KCC2 as well as a similar distribution of them in the outer and inner plexiform layers (OPL and IPL) in retinal sections at P23. Functionally, GABA A receptor and KCC2 are both membrane proteins, closely related, which modulate GABAergic inhibition. An upregulation of *Slc12a5* expression could increase Cl- extrusion, and subsequent Cl− gradient through the membrane facilitating the entry of Cl− through the ionotropic GABA A receptor. In addition, we showed an upregulation of *Gabra1*, which could exacerbate the inhibition induced by GABA through its binding to GABA A receptors. The higher staining of KCC2 and GABARα1 observed in the inner retina of rd10 mice (Fig. 6B, C) would support this idea. In addition, it has been suggested that the remodeling of the inner retina is accompanied by upregulation of glycine and GABA A receptors in *rd1* mice [81]. Our findings suggest that therapeutic approaches aimed at reducing GABAergic inhibition and increasing the excitation of RGCs may be beneficial for RP. It has been suggested that anticonvulsants, such as tiagabine and vigabatrin, have a protective role in light-induced retinal degeneration in *Abca4*^*−/−*^*Rdh8*^*−/−*^ mice, and that this protection is mediated by their role as GABA modulators [82]. Novel antiepileptic drugs act selectively through the GABAergic system and, although the underlying mechanism relating antiepileptic drugs with retinal degeneration is not yet fully explored, our model highlights the role of anticonvulsants in the mechanistic map of RP, probably through GABA-related processes [83]. Digging into the drugs targeting relevant predictive KDTs, the overrepresentation of anesthetic, hypnotics, and sedative drugs such as Pentazocine, Ketamine, Taurine, Flumazenil, and Phenobarbital among other drugs predicted by our model, coherently remark the relation between the anti-excitotoxic effects of certain anesthetics and their neuroprotection that has been long studied [84], whilst its role (and potential use) in RP remains undiscovered [85]. As a matter of fact, selected predicted drugs shown in Fig. 7C share a neuroprotective effect, also appearing to be related to fatty acid processes. Omega 3-related compounds, also predicted by our model, appear to have an impact on the progression of RP as discussed in Olivares-Gonzalez et al. [44].

The effectiveness of applying mechanistic models and ML methodologies to drug repurposing has been undoubtedly demonstrated in several works [17, 19, 86]. These approaches allow for the virtual testing of hundreds to thousands of scenarios with very minimal resources. While many ML-based repurposing strategies focus on the physicochemical properties of the drugs [87], they often overlook the overall impact on diseases or cells. This is where mechanistic models of disease come into play, offering insights not only into disease mechanisms but also into drug actions in specific disease contexts [88]. Indeed, supervised ML has been successfully used to boost how these tools model cellular behavior, improving the accuracy of mechanistic models in characterizing cell types [14, 49], identifying targets for disease treatments [17, 42], or predicting the effect of single or combined drugs on cells [50, 51].

The main purpose of this work was to detect actionable target genes that could have an impact on RP, providing an explorable resource of knowledge. Nevertheless, this methodology can also be used to model all cellular mechanisms to evaluate potential undesired off-target and side effects as well as to the identification of new disease-related genes, such as *GABRA*, *GRIN1*, *KCC* or *SLC12A5* whose mutations can lead to the development of retinal dystrophies. Due to its exploratory nature, the lack of ground truth in the validation process comes as a limitation of the method, we have demonstrated the robustness of the results experimentally validating the role of selected KDTs, with no previous association with RP, in RP murine models. However, further research is needed to assess the in vitro and in vivo impact that the drugs targeting relevant KDTs would have on the disease progression or initiation.

Nevertheless, given that RP has more than 250 genes identified, but only 50% of RP cases are attributable to identified genes, this application on its own has tremendous potential for the clinical community, as a tool to evaluate the impact of genes of uncertain significance [5].

## Conclusions

In this work, an actionable map of Retinitis Pigmentosa (RP) is presented, reflecting the mechanisms and dynamics of the functions involved in disease initiation and progression. The map serves as a resource for further analysis, validations, and computational modeling, such as network or graph analyses. The methodology identifies potential targets impacting RP processes, providing a list of relevances and pinpointing the mechanism, function, and circuit affected as well as the KDT responsible for the predicted impact. These relevances can guide and aid in finding possible clinical intervention points. Although focused on signaling pathways, the model can be expanded to other types of pathways using directed interaction networks, and various datasets representing gene/protein abundance, bearing in mind that limitations in sample numbers and balanced dimensionality must be considered. The model’s strength lies in its applicability to scenarios with limited knowledge, such as rare or emerging diseases like COVID-19 [19, 21, 86]. It can be adapted to infer interactions between genes and mechanisms, providing a sophisticated way to model cell behavior under disease conditions. Combining mechanistic models with ML provides a useful tool to explore disease druggable space, mechanisms, and pathogenesis aiding in genetic diagnosis and identifying genes with a key role in diseases. The possibility of using the available genomic repositories to formulate hypotheses and extrapolate conclusions in the rare diseases field constitutes an attractive opportunity to guide and accelerate the experimental validations in order to focus and prioritize target candidates.

### Supplementary Information

**Additional file 1: Table S1.** Table of number of genes, and genes in KEGG signaling pathways contained in HiPathia R package sharing an increasing number of RP-HPO terms.

**Additional file 2: Table S2.** Table of RP mechanistic map circuits with their code, name (Pathway: effector node), their hallmark/s module/s and the UniprotKB and Gene Ontology functions tagging them.

**Additional file 3: Table S3.** Table with DrugBank 5.1.8 database filtered by approved human drugs with known pharmacological action and known protein targets.

**Additional file 4: Table S4.** Table with SHAP score of all KDT (columns) over all RP mechanistic map circuits (rows).

**Additional file 5: Table S5.** Table with selection of predictive KDT (columns) over all RP mechanistic map circuits (rows) in Boolean format (1 = selected; 0 = not selected).

**Additional file 6: Table S6.** Table with machine learning model performance results (stability and R^2^ metrics) over both the whole RP mechanistic map and the per specific circuit models.

**Additional file 7: Table S7.** Table with relevant predictive KDTs and their clusters obtained by hierarchical clustering of SHAP scores (sheet 1). Cluster 1 Panther functional over-representation analysis of GO molecular function and GO biological process (sheet 2). Cluster 2 Panther functional over-representation analysis of GO molecular function and GO biological process (sheet 3). Cluster 3 Panther functional over-representation analysis of GO molecular function and GO biological process (sheet 4).

**Additional file 8: Table S8.** Validated genes quantification by qRT-PCR, normalized to the values of control mice (100%).

**Additional file 9: Table S9.** Table of relevant predictive KDTs with their corresponding drug and pharmacological actions.

**Additional file 10: Table S10.** Over-represented categories from the different ATC levels tagging drugs targeting relevant predictive KDTs.

**Additional file 11: Figure S1.** Composition and functional description of the Retinitis Pigmentosa Map. A) Stacked bar-plot representing the number of genes added to the RP-genes found in ORPHANET/OMIM (blue) from the total number of genes sharing RP-HPO terms (red) in signaling pathways (Y-axis) vs the number of shared RP-HPO terms (X-axis). B) Balloon plot illustrating the percentage, as a color gradient, of the RP-hallmarks (X-axis) represented in each of the 40 KEGG signaling pathways composing the RP Map (Y-axis). The size of the balloon represents the nº of circuits of each pathway. C) Radar plot representing the percentage of the RP map (number of circuits with hallmark tagged/total number of circuits) annotated by each hallmark taking into account that a circuit might be tagged by more than one hallmark.

**Additional file 12: Figure S2.** Profiling and functional analysis of drug target clusters predicted as relevant. A) GAP statistic method metrics for calculating the optimal number of drug target (KDT) clusters, based on their normalized SHAP scores (Gap Statistics chooses the number of clusters where the biggest jump in within-cluster distance occurred). B) Distribution of the SHAP score values of the 109 relevant KDTs on a t-SNE, colored by cluster. C) Gene Ontology (GO) enrichment analysis of significant GO Biological Process and Molecular function terms obtained for the three KDT clusters (cluster 1 in red, cluster 2 in yellow and cluster 3 in green), colored by Fold enrichment.

**Additional file 13: Figure S3.** Heatmap plot of the normalized SHAP scores from the 109 predicted drug targets (KDTs) (X-axis) over the 209 stable circuits (Y-axis) of the Retinitis Pigmentosa Map. The sign indicates the direction of the KDT influence over each specific circuit and the score value depicts how strong is the influence of a specific KDT for predicting the activity of a specific circuit. The top color bar represents the most frequent drug effects of the drugs targeting that specific KDT.

**Additional file 14: Figure S4.** Augmented histological study of selected KDTs in retinas sections of rd10 mice, murine model of Retinitis Pigmentosa. Confocal micrographs at a 63× augmentations of another set of transverse sections through rd10 mice retina. DAPI nuclear staining illustrating the retinal cytoarchitecture (retinal inner nuclear layer INL, retinal inner plexiform layer IPL, retinal outer nuclear layer ONL, retinal outer plexiform layer OPL and retinal ganglion cell layer GCL). Photoreceptor degeneration (A, B), and retinal distribution of selected KDTs KCC2 (C, D), GABARα1 (E, F) and NR1 (G, H) in control and rd10 mice at P23.

**Additional file 15.** Additional Methods: Detailed information in Machine Learning methodology and in material and methods of experimental assays performed.

## Data Availability

Gene expression data was downloaded from the GTEx Portal (GTEx Analysis Release V8; dbGaP Accession phs000424.v8.p2) [31]. Drug-target interactions were extracted from the DrugBank DB release 5.1.8 [33]. The complete RP mechanistic map can be explored at: http://hipathia.babelomics.org/RP_Mechanistic_Map/ [22]. All the code used for the method and the analyses is publicly available. The code for the RP-specific workflow can be found at: https://github.com/babelomics/drexml-retinitis/releases/tag/v1.0.0 [89].

## Abbreviations

*Alox5*: Arachidonate 5-lipoxygenase (mouse)
ATC: Anatomical Therapeutic Chemical Classification
B2M: Beta2-microglobulin
BCA: Bicinchoninic acid
BSA: Bovine serum albumin
cAMP: Cyclic adenosine monophosphate
CIPF: Research Center Principe Felipe
COVID19: Coronavirus disease-19
CPU: Central processing unit
DB: Database
*Elovl4*: Fatty acid elongase 4 (mouse)
ESI: Electrospray ionization
EU: European Union
FDR: False discovery rate
FGF2: Fibroblast growth factor 2 (human)
GABA: Gamma-aminobutyric acid
GABRA1 or GABARα1: Alpha-1 (α1) subunit of the GABA A receptor protein: Alpha-1 (α1) subunit of the GABA A receptor protein
*Gabra1*α1: Subunit of the GABAA receptor (mouse)
*Gabre*: Epsilon subunit of GABAA receptor (mouse)
GCL: Ganglion cell layer (retina)
*Glra2*: Glycine receptor alpha 2 (mouse)
GO: Gene Ontology
GPU: Graphics processing unit
GRIN1: Glutamate ionotropic receptor NMDA type subunit 1 (human)
GTEx: Genotype-tissue expression project
HIF-1: Hypoxia inducible factor 1
HkI: Hexokinase 1(mouse)
HPLC–MS: High performance liquid chromatography–mass spectrometry
HPO: Human Phenotype Ontology
HRH3: Histamine receptor H3 (human)
INL: Inner nuclear layer (retina)
IPL: Inner plexiform layer (retina)
IVC: Individually ventilated cages
KCC2: K+/Cl− cotransporter 2
KDT: Known drug target
KEGG: Kyoto Encyclopedia of Genes and Genomes
LC–MS: Liquid chromatography–mass spectrometry
LCA: Leber congenital amaurosis
ML: Machine learning
MORF: Multi-output Random Forest
MPA: Mechanistic pathway analysis
MRM: Multiple reaction monitoring
MSE: Mean squared error
NMDA: *N*-Methyl-d-aspartate (human)
NMF: Nonnegative matrix factorization
ONL: Outer nuclear layer (retina)
OPL: Outer plexiform layer (retina)
ORA: Over-representation analysis
PCR: Polymerase chain reaction
PMSF: Phenylmethylsulfonyl fluoride
PVDF: Polyvinylidene difluoride
RD1: Retinal degeneration 1
RD10: Retinal degeneration 10
RIPA: Radioimmunoprecipitation assay
RGC: Retinal ganglion cells
ROCK1: Rho associated coiled-coil containing protein kinase 1 (human)
RP: Retinitis pigmentosa
SEM: Standard error of mean
SDS: Sodium dodecyl sulfate
SHAP: Shapley additive explanations
SLC12A5: Solute carrier family 12 member 5 (human)
TAS: Traceable author statement
TBST: Tris-buffered saline-Tween
TMM: Trimmed mean of M values

## References

[1] Ferrari S, Di Iorio E, Barbaro V, Ponzin D, Sorrentino FS, Parmeggiani F (2011). Retinitis pigmentosa: genes and disease mechanisms. Curr Genom.

[2] Parmeggiani F (2011). Clinics, epidemiology and genetics of retinitis pigmentosa. Curr Genom.

[3] Chang AY, Tsang SH, Quinn PMJ (2023). Challenges of treatment methodologies and the future of gene therapy and stem cell therapy to treat retinitis pigmentosa. Retinitis pigmentosa.

[4] Daiger SP, Sullivan LS, Bowne SJ (2013). Genes and mutations causing retinitis pigmentosa. Clin Genet.

[5] Sorrentino FS, Gallenga CE, Bonifazzi C, Perri P (2016). A challenge to the striking genotypic heterogeneity of retinitis pigmentosa: a better understanding of the pathophysiology using the newest genetic strategies. Eye.

[6] Stephens ZD, Lee SY, Faghri F, Campbell RH, Zhai C, Efron MJ (2015). Big data: astronomical or genomical?. PLOS Biol.

[7] Boycott KM, Hartley T, Biesecker LG, Gibbs RA, Innes AM, Riess O (2019). A diagnosis for all rare genetic diseases: the horizon and the next frontiers. Cell.

[8] Henrie A, Hemphill SE, Ruiz-Schultz N, Cushman B, DiStefano MT, Azzariti D (2018). ClinVar miner: demonstrating utility of a web-based tool for viewing and filtering ClinVar data. Hum Mutat.

[9] Yu MK, Kramer M, Dutkowski J, Srivas R, Licon K, Kreisberg J (2016). Translation of genotype to phenotype by a hierarchy of cell subsystems. Cell Syst.

[10] Amadoz A, Sebastian-Leon P, Vidal E, Salavert F, Dopazo J (2015). Using activation status of signaling pathways as mechanism-based biomarkers to predict drug sensitivity. Sci Rep.

[11] Salavert F, Hidago MR, Amadoz A, Çubuk C, Medina I, Crespo D (2016). Actionable pathways: interactive discovery of therapeutic targets using signaling pathway models. Nucleic Acids Res.

[12] Hidalgo MR, Cubuk C, Amadoz A, Salavert F, Carbonell-Caballero J, Dopazo J (2017). High throughput estimation of functional cell activities reveals disease mechanisms and predicts relevant clinical outcomes. Oncotarget.

[13] Cubuk C, Hidalgo MR, Amadoz A, Pujana MA, Mateo F, Herranz C (2018). Gene expression integration into pathway modules reveals a pan-cancer metabolic landscape. Cancer Res.

[14] Falco MM, Peña-Chilet M, Loucera C, Hidalgo MR, Dopazo J (2020). Mechanistic models of signaling pathways deconvolute the glioblastoma single-cell functional landscape. NAR Cancer.

[15] Peña-Chilet M, Esteban-Medina M, Falco MM, Rian K, Hidalgo MR, Loucera C (2019). Using mechanistic models for the clinical interpretation of complex genomic variation. Sci Rep.

[16] Razzoli M, Frontini A, Gurney A, Mondini E, Cubuk C, Katz LS (2016). Stress-induced activation of brown adipose tissue prevents obesity in conditions of low adaptive thermogenesis. Mol Metab.

[17] Esteban-Medina M, Peña-Chilet M, Loucera C, Dopazo J (2019). Exploring the druggable space around the Fanconi anemia pathway using machine learning and mechanistic models. BMC Bioinform.

[18] Loucera C, Esteban-Medina M, Rian K, Falco MM, Dopazo J, Peña-Chilet M (2020). Drug repurposing for COVID-19 using machine learning and mechanistic models of signal transduction circuits related to SARS-CoV-2 infection. Signal Transduct Target Ther.

[19] Çubuk C, Can FE, Peña-Chilet M, Dopazo J (2020). Mechanistic models of signaling pathways reveal the drug action mechanisms behind gender-specific gene expression for cancer treatments. Cells.

[20] Hidalgo MR, Amadoz A, Çubuk C, Carbonell-Caballero J, Dopazo J (2018). Models of cell signaling uncover molecular mechanisms of high-risk neuroblastoma and predict disease outcome. Biol Direct.

[21] Montanuy H, Martínez-Barriocanal Á, Antonio Casado J, Rovirosa L, Ramírez MJ, Nieto R (2020). Gefitinib and afatinib show potential efficacy for Fanconi anemia-related head and neck cancer. Clin Cancer Res Off J Am Assoc Cancer Res.

[22] Loucera C, Peña-Chilet M, Esteban-Medina M, Muñoyerro-Muñiz D, Villegas R, Lopez-Miranda J (2021). Real world evidence of calcifediol or vitamin D prescription and mortality rate of COVID-19 in a retrospective cohort of hospitalized Andalusian patients. Sci Rep.

[23] Loucera C, Carmona R, Esteban-Medina M, Bostelmann G, Muñoyerro-Muñiz D, Villegas R (2023). Real-world evidence with a retrospective cohort of 15,968 COVID-19 hospitalized patients suggests 21 new effective treatments. Virol J.

[24] Ashburn TT, Thor KB (2004). Drug repositioning: identifying and developing new uses for existing drugs. Nat Rev Drug Discov.

[25] Simoens S, Cassiman D, Dooms M, Picavet E (2012). Orphan drugs for rare diseases: is it time to revisit their special market access status?. Drugs.

[26] Pushpakom S, Iorio F, Eyers PA, Escott KJ, Hopper S, Wells A (2019). Drug repurposing: progress, challenges and recommendations. Nat Rev Drug Discov.

[27] Orphanet: an online database of rare diseases and orphan drugs. Copyright, INSERM 1997. http://www.orpha.net. Accessed 16 Nov 2022.

[28] Hamosh A, Scott AF, Amberger JS, Bocchini CA, McKusick VA (2005). Online Mendelian Inheritance in Man (OMIM), a knowledgebase of human genes and genetic disorders. Nucleic Acids Res.

[29] Köhler S, Gargano M, Matentzoglu N, Carmody LC, Lewis-Smith D, Vasilevsky NA (2021). The human phenotype ontology in 2021. Nucleic Acids Res.

[30] Kanehisa M, Goto S, Sato Y, Kawashima M, Furumichi M, Tanabe M (2014). Data, information, knowledge and principle: back to metabolism in KEGG. Nucleic Acids Res.

[31] Lonsdale J, Thomas J, Salvatore M, Phillips R, Lo E, Shad S (2013). The genotype-tissue expression (GTEx) project. Nat Genet.

[32] Chen Y, Lun A, McCarthy D, Zhou X, Robinson M, Smyth G. edgeR: empirical analysis of digital gene expression data in R. 2019. http://bioinf.wehi.edu.au/edgeR.

[33] Wishart DS, Feunang YD, Guo AC, Lo EJ, Marcu A, Grant JR (2018). DrugBank 5.0: a major update to the DrugBank database for 2018. Nucleic Acids Res.

[34] Hidalgo MR. hipathia: HiPathia: high-throughput pathway analysis. 2019.

[35] Retinitis pigmentosa mechanistic map 2023 viewer. http://hipathia.babelomics.org/RP_Mechanistic_Map/.

[36] Hidalgo MR, Carbonell-Caballero J, Salavert F, Amadoz A, Cubuk Ç, Dopazo J. hipathia: HiPathia: high-throughput pathway analysis. Bioconductor version: release (3.15). 2022. https://bioconductor.org/packages/hipathia/. Accessed 22 June 2022.

[37] Segal M, Xiao Y (2011). Multivariate random forests. WIREs Data Min Knowl Discov.

[38] Lundberg SM, Lee SI, Guyon I, Luxburg UV, Bengio S, Wallach H, Fergus R, Vishwanathan S (2017). A unified approach to interpreting model predictions. Advances in neural information processing systems 30.

[39] Lundberg SM, Erion G, Chen H, DeGrave A, Prutkin JM, Nair B (2020). From local explanations to global understanding with explainable AI for trees. Nat Mach Intell.

[40] Nogueira S, Sechidis K, Brown G (2018). On the stability of feature selection algorithms. J Mach Learn Res.

[41] Gaujoux R, Seoighe C (2010). A flexible R package for nonnegative matrix factorization. BMC Bioinform.

[42] Mi H, Muruganujan A, Casagrande JT, Thomas PD (2013). Large-scale gene function analysis with PANTHER classification system. Nat Protoc.

[43] Liu W, Liu S, Li P, Yao K (2022). Retinitis pigmentosa: progress in molecular pathology and biotherapeutical strategies. Int J Mol Sci.

[44] Olivares-González L, Velasco S, Gallego I, Esteban-Medina M, Puras G, Loucera C (2022). An SPM-enriched marine oil supplement shifted microglia polarization toward M2, ameliorating retinal degeneration in rd10 mice. Antioxidants.

[45] Fricker M, Tolkovsky AM, Borutaite V, Coleman M, Brown GC (2018). Neuronal cell death. Physiol Rev.

[46] Sancho-Pelluz J, Arango-Gonzalez B, Kustermann S, Romero FJ, van Veen T, Zrenner E (2008). Photoreceptor cell death mechanisms in inherited retinal degeneration. Mol Neurobiol.

[47] Strasser A, O’Connor L, Dixit VM (2000). Apoptosis signaling. Annu Rev Biochem.

[48] Murakami Y, Notomi S, Hisatomi T, Nakazawa T, Ishibashi T, Miller JW (2013). Photoreceptor cell death and rescue in retinal detachment and degenerations. Prog Retin Eye Res.

[49] Murakami Y, Matsumoto H, Roh M, Giani A, Kataoka K, Morizane Y (2014). Programmed necrosis, not apoptosis, is a key mediator of cell loss and DAMP-mediated inflammation in dsRNA-induced retinal degeneration. Cell Death Differ.

[50] Yoshida N, Ikeda Y, Notomi S, Ishikawa K, Murakami Y, Hisatomi T (2013). Clinical evidence of sustained chronic inflammatory reaction in retinitis pigmentosa. Ophthalmology.

[51] Chu WM (2013). Tumor necrosis factor. Cancer Lett.

[52] Cullen SP, Henry CM, Kearney CJ, Logue SE, Feoktistova M, Tynan GA (2013). Fas/CD95-induced chemokines can serve as “find-me” signals for apoptotic cells. Mol Cell.

[53] Olivares-González L, Martínez-Fernández de la Cámara C, Hervás D, Millán JM, Rodrigo R (2018). HIF-1α stabilization reduces retinal degeneration in a mouse model of retinitis pigmentosa. FASEB J.

[54] Martínez-Fernández de la Cámara C, Olivares-González L, Hervás D, Salom D, Millán JM, Rodrigo R (2014). Infliximab reduces Zaprinast-induced retinal degeneration in cultures of porcine retina. J Neuroinflamm.

[55] Ting AT, Bertrand MJM (2016). More to life than NF-κB in TNFR1 signaling. Trends Immunol.

[56] Van Herreweghe F, Festjens N, Declercq W, Vandenabeele P (2010). Tumor necrosis factor-mediated cell death: to break or to burst, that’s the question. Cell Mol Life Sci.

[57] Olivares-González L, Velasco S, Millán JM, Rodrigo R (2020). Intravitreal administration of adalimumab delays retinal degeneration in rd10 mice. FASEB J.

[58] Najjar M, Saleh D, Zelic M, Nogusa S, Shah S, Tai A (2016). RIPK1 and RIPK3 kinases promote cell-death-independent inflammation by toll-like receptor 4. Immunity.

[59] Moriwaki K, Chan FKM (2017). The inflammatory signal adaptor RIPK3: functions beyond necroptosis. Int Rev Cell Mol Biol.

[60] Abais JM, Xia M, Zhang Y, Boini KM, Li PL (2015). Redox regulation of NLRP3 inflammasomes: ROS as trigger or effector?. Antioxid Redox Signal.

[61] Totsuka K, Ueta T, Uchida T, Roggia MF, Nakagawa S, Vavvas DG (2019). Oxidative stress induces ferroptotic cell death in retinal pigment epithelial cells. Exp Eye Res.

[62] Gil J, Almeida S, Oliveira CR, Rego AC (2003). Cytosolic and mitochondrial ROS in staurosporine-induced retinal cell apoptosis. Free Radic Biol Med.

[63] Tajiri S, Oyadomari S, Yano S, Morioka M, Gotoh T, Hamada JI (2004). Ischemia-induced neuronal cell death is mediated by the endoplasmic reticulum stress pathway involving CHOP. Cell Death Differ.

[64] Eijkelkamp N, Linley JE, Baker MD, Minett MS, Cregg R, Werdehausen R (2012). Neurological perspectives on voltage-gated sodium channels. Brain J Neurol.

[65] Das S, Chen Y, Yan J, Christensen G, Belhadj S, Tolone A (2021). The role of cGMP-signalling and calcium-signalling in photoreceptor cell death: perspectives for therapy development. Pflüg Arch Eur J Physiol.

[66] Zamponi GW, Striessnig J, Koschak A, Dolphin AC (2015). The physiology, pathology, and pharmacology of voltage-gated calcium channels and their future therapeutic potential. Pharmacol Rev.

[67] Ishikawa M (2013). Abnormalities in glutamate metabolism and excitotoxicity in the retinal diseases. Scientifica.

[68] Duan F, Xiao Z, Wang Y, Sun X, Tang Z, Wang R (2022). Metabolic alterations in the visual pathway of retinitis pigmentosa rats: a longitudinal multimodal magnetic resonance imaging study with histopathological validation. NMR Biomed.

[69] Jones BW, Pfeiffer RL, Ferrell WD, Watt CB, Marmor M, Marc RE (2016). Retinal remodeling in human retinitis pigmentosa. Exp Eye Res.

[70] Pfeiffer RL, Marc RE, Jones BW (2020). Persistent remodeling and neurodegeneration in late-stage retinal degeneration. Prog Retin Eye Res.

[71] Marc RE, Jones BW, Watt CB, Strettoi E (2003). Neural remodeling in retinal degeneration. Prog Retin Eye Res.

[72] Caravaca-Rodriguez D, Gaytan SP, Suaning GJ, Barriga-Rivera A (2022). Implications of neural plasticity in retinal prosthesis. Invest Ophthalmol Vis Sci.

[73] Kolb H, Fernandez E, Nelson R (1995). Webvision: the organization of the retina and visual system.

[74] Rivera C, Voipio J, Payne JA, Ruusuvuori E, Lahtinen H, Lamsa K (1999). The K+/Cl− co-transporter KCC2 renders GABA hyperpolarizing during neuronal maturation. Nature.

[75] Popova E (2014). Ionotropic GABA receptors and distal retinal ON and OFF responses. Scientifica.

[76] Tan KR, Rudolph U, Lüscher C (2011). Hooked on benzodiazepines: GABAA receptor subtypes and addiction. Trends Neurosci.

[77] Pan F, Toychiev A, Zhang Y, Atlasz T, Ramakrishnan H, Roy K (2016). Inhibitory masking controls the threshold sensitivity of retinal ganglion cells. J Physiol.

[78] Jensen RJ, Rizzo JF (2011). Effects of GABA receptor antagonists on thresholds of P23H rat retinal ganglion cells to electrical stimulation of the retina. J Neural Eng.

[79] Jensen RJ (2012). Blocking GABA(C) receptors increases light responsiveness of retinal ganglion cells in a rat model of retinitis pigmentosa. Exp Eye Res.

[80] Wang Q, Banerjee S, So C, Qiu C, Lam HIC, Tse D (2020). Unmasking inhibition prolongs neuronal function in retinal degeneration mouse model. FASEB J.

[81] Srivastava P, Sinha-Mahapatra SK, Ghosh A, Srivastava I, Dhingra NK (2015). Differential alterations in the expression of neurotransmitter receptors in inner retina following loss of photoreceptors in rd1 mouse. PLoS ONE.

[82] Schur RM, Gao S, Yu G, Chen Y, Maeda A, Palczewski K (2018). New GABA modulators protect photoreceptor cells from light-induced degeneration in mouse models. FASEB J.

[83] Czapiński P, Blaszczyk B, Czuczwar SJ (2005). Mechanisms of action of antiepileptic drugs. Curr Top Med Chem.

[84] Olney JW, Price MT, Fuller TA, Labruyere J, Samson L, Carpenter M (1986). The anti-excitotoxic effects of certain anesthetics, analgesics and sedative-hypnotics. Neurosci Lett.

[85] Iwata M, Inoue S, Kawaguchi M, Furuya H (2012). Effects of diazepam and flumazenil on forebrain ischaemia in a rat model of benzodiazepine tolerance. Br J Anaesth.

[86] Loucera C, Esteban-Medina M, Rian K, Falco MM, Dopazo J, Peña-Chilet M (2020). Drug repurposing for COVID-19 using machine learning and mechanistic models of signal transduction circuits related to SARS-CoV-2 infection. Signal Transduct Target Ther.

[87] Choudhury C, Arul Murugan N, Priyakumar UD (2022). Structure-based drug repurposing: traditional and advanced AI/ML-aided methods. Drug Discov Today.

[88] Ostaszewski M, Niarakis A, Mazein A, Kuperstein I, Phair R, Orta-Resendiz A (2021). COVID19 disease map, a computational knowledge repository of virus-host interaction mechanisms. Mol Syst Biol.

[89] Drug repositioning modelization of retinitis pigmentosa with explainable machine learning. https://github.com/babelomics/drexml-retinitis/releases/tag/v1.0.0.

