# Supplementary material for: The mechanistic functional landscape of retinitis pigmentosa: a machine learning-driven approach to therapeutic target discovery
Source: J Transl Med. 2024 Feb 6;22:139. doi: 10.1186/s12967-024-04911-7 (PMC10848380; doi:10.1186/s12967-024-04911-7)
Supplement: Supplementary file 8 — Additional file 8: Table S8. Validated genes quantification by qRT-PCR, normalized to the values of control mice (100%). [file 12967_2024_4911_MOESM8_ESM.docx]

Additional materials for “The mechanistic functional Landscape of Retinitis Pigmentosa: a Machine Learning-driven approach to drug Repurposing”

# Additional methods

## Machine Learning

In this section, we provide additional details on the machine learning procedure for Known Drug Target (KDT) selection and prioritization in the context of the functional map of Retinitis Pigmentosa.

### Explainable Machine Learning Model

The explainable machine learning model is a feature selection and ranking method that aims to elucidate which drug targets (KDTs) could be potential regulators of a given disease map. The procedure takes as input sample-wise paired normalized gene expression and circuit activity matrices of shapes [n_samples, n_kdts] and [n_samples, n_circuits], respectively. The output consists of two matrices: (i) a sparse matrix of shape [n_kdts, n_circuits], which contains the signed relevance of each KDT concerning the circuits that configure the disease map, and (ii) a results matrix that summarizes the generalization performance of the model across the disease map.

The method leverages the use of (i) a Multi-output Random Forest [(1)](https://www.zotero.org/google-docs/?TC937w), the learner, to establish a map between the gene and signaling spaces, (ii) the SHapley Additive exPlanations (SHAP values) [(2)](https://www.zotero.org/google-docs/?oSUEfX) to rank each gene-circuit pair, (iii) a feature selection procedure based on pairing the SHAP rankings with the predictive power of the learner, and (iv) a validation schema to evaluate the performance at the predictive and feature selection levels.

### Feature selection and ranking

Given a set of training samples (X, Y), where X contains the gene expression values of each KDT, and Y the paired signaling activity for each circuit of the disease map, the method can be decomposed into the following steps:

1. Randomly subsample the training samples into the background (0.7 the sample size) and validation (0.3 the sample size) sets.
2. Fit the learner to the background set and compute the multivariate R² score of the validation set.
3. Explain the fitted learner by computing the tree-accelerated SHAP values [(3)](https://www.zotero.org/google-docs/?Ep6LHs) for each validation sample, gene, and circuit, resulting in a tensor V of shape [n_samples_validation, n_kdts, n_circuits], which is summarized across the sample axis using the mean absolute value. Thus, we end with a matrix R of [n_genes, n_circuits] where cell (i, j) contains the relevance for the gene i concerning the circuit j. To compute the signed relevance of element (i, j) the method extracts the sign of the coefficient of a linear model that regresses the expression vector of gene i for the vector s_i,j that contains the SHAP values of gene i concerning circuit j, ending with a signed relevance matrix S of shape [n_genes, n_cirucits]. Note that for a fixed circuit j, the absolute relevances {s_ij: i=1, …, n_genes} establish a ranking for circuit j, where the top-ranked KDTs indicate potential regulators of circuit j.
4. To select the most relevant genes for a given circuit j, the model uses the q_th quantile of the absolute relevances filtered by the R² score at the j component (R²_j) using the following convex formula: q_j = q_th + ((1 - R²_j) / (1 - R²_th)) * (1 - q_th). Therefore, when the circuit is perfectly predicted (R²_j=1) and q_th=0.95, the model selects the top 5% genes as ranked by the mean absolute Shapley value for circuit j. In contrast, as the predictability decreases so does the percentage of genes selected (the case R²_j <= R2_th implies no selected genes for circuit j).
5. For each circuit j, the model selects those KDT genes ranked above the q_j quantile, represented by an indicator matrix I of shape [n_genes, n_circuits]. Finally, the model summarizes the relevance and indicator matrices by computing the element-wise (Hadamard) product of S and I to obtain the final result.

### Validation schema

Two quality measures are used to validate the performance of the machine learning method from a data-driven point of view: on the one hand, the R² score is used to quantify the quality of the mapping between the gene and signaling spaces, and, on the other hand, the Nogueira stability test [(4)](https://www.zotero.org/google-docs/?B2Tvhv) is used to ensure the consistency of the feature selection procedure. To control the variability of the measures we follow the resampling strategy proposed by Nogueira et al., 2018 [(4)](https://www.zotero.org/google-docs/?1bYu6D).

The resampling strategy consists of splitting the samples into training and test sets using half the sample size for each set, fitting the proposed model to the training set, refitting the learner to the training set using the features selected by the model, and assessing its performance using the R² score of the test set. The resampling is performed in 100 bootstrap samples of the dataset. The model fit and scoring strategy is repeated for each train/test partition, resulting in a distribution of R² scores (one score for each test fold) and a selected gene population (one selection per training fold). Finally, we report the point estimates and 95% confidence intervals for the R² and stability measures, one per circuit, and a map-wise average.

The Nogueira stability test is a reliable measure of the stability of feature selection algorithms. It is based on a small set of generic properties that describe desirable behaviors of a stability measure and has three key properties: consistency, strict monotonicity, and bounded. This makes it possible to conduct confidence intervals and hypothesis tests on the stability, allowing for rigorous experimental comparison of feature selection algorithms. The Nogueira stability test has several beneficial properties that make it stand out from previous measures, making it possible to estimate the stability of features dependably.

Consistency refers to the stability estimator being consistent, concerning data sampling the inherent stochasticity, and converging to the true stability value as the sample size increases. Strict monotonicity means that the stability estimator should be a strictly decreasing function of the sample variances of the variables. Bounded refers to the stability estimator being upper/lower bounded by constants not dependent on the sample size.

**Stability estimate:** The stability estimate, s, ranges from 0 to 1 and measures the robustness of the feature selection method. A stability estimate of 1 indicates that the method always selects the same features, while a stability estimate of 0 indicates that the method selects features randomly. A stability estimate of less than 0.4 is considered poor, a stability estimate between 0.4 and 0.75 is considered good, and a stability estimate greater than or equal to 0.75 is considered excellent.

**R-squared score:** The R-squared score, also known as the coefficient of determination, ranges from -infinity to 1. A score of 0 indicates that the model is no better than predicting the mean, and a score of -infinity implies that the model is arbitrarily wrong.

### Hyperparameters

Thanks to the continuous improvements in our drug repurposing methodology [(5–7)](https://www.zotero.org/google-docs/?SPkxEF) we have found a set of hyperparameters that allows the model to replicate the results of our previous experiments while providing the latest advances of the tool (per-circuit relevance and stability measures, sparser explanations and parallelized GPU/CPU explanations). As a result, the learner hyperparameters are set to 200 multi-output regression trees with a maximum depth of 8, which are fitted over partitions of a maximum of *ceil(sqrt(n_kdts) + 20)* genes. The mean squared error (*MSE*) is used as the criterion to elucidate the quality of any given split. The SHAP values are integrated using a subsample (n=1000) of the background set to accelerate the process, following the Python shap package (v 0.41) recommendations. The feature selection hyperparameters are set to an R² threshold (*R^2_th*) of 0.5, and a quantile threshold (*q_th*) of 0.95.

### Software implementation

In <https://github.com/babelomics/drexml-retinitis/releases/tag/v1.0.1> we provide a workflow that can reproduce the results presented in the main manuscript. Note that, by default, the software downloads pre-computed matrices for the GTEx samples stored in a Zenodo repository [(8)](https://www.zotero.org/google-docs/?wtOsBV) along with the other databases required to reproduce the results. Additionally, the GitHub repository provides some recommendations to update the workflow in case anyone wants to reanalyze our approach for drug repurposing in Retinitis Pigmentosa (e.g. when new versions of the databases are released).

To reproduce the results the only requirement is a Linux x64 system able to run/install conda. Everything has been tested using a Linux x64 system (Ubuntu 20.04, 22.04, 23.04, and a SLURM-based Rocky Linux 8.5 system) with the Miniconda v3 distribution as the packaging system.

The repository contains YML conda specification files to reproduce the Python and R environments required to run the workflow. Part of the workflow can be accelerated using any number of CUDA devices (CUDA version < 12) by forcing the compilation of the GpuTreeShap CUDA extensions [(9)](https://www.zotero.org/google-docs/?1HO5kl) that are part of the SHAP Python package. CUDA devices with more than 16 GB of RAM are recommended.

### Animal model of Retinitis Pigmentosa

*Rd10* mice were used as a mouse model of autosomal recessive RP. The Pde6brd10/rd10 (*rd10*) mouse is a very well-characterized animal model for RP. *rd10* mice present a mutation at the C-terminal end of the phosphodiesterase 6b (*Pde6b*) gene (exon 13), which encodes the β subunit of rod phosphodiesterase. Briefly, this mutation prevents the cyclic guanosine-monophosphate (cGMP) hydrolysis, the cGMP-dependent channels remain opened, cation influx increases, phototransduction does not occur properly and eventually rod photoreceptors degenerate and later cone photoreceptor as well [(10)](https://www.zotero.org/google-docs/?HdywPk). In our housing conditions, we have previously observed a first peak of photoreceptor degeneration at postnatal day (P)18 and a second peak of photoreceptor loss at P60 (*rd10* mouse is a good tool to study this retinal degeneration. The C57Bl/6J mice are wild-type animals with the same genetic background as the *rd10* mice and therefore were used as a control group. Mice were kept under a 12 h light/dark cycle, with humidity and temperature controlled. Mothers were fed with a standard chow diet and water *ad libitum*. All cages were placed on the lower shelf of an IVC rack with light illuminance of 115 ± 7 lux (95% CI: 98–131). Mice were housed in the Animal Facility of Research Center Principe Felipe (CIPF). This study was carried out following the European Union Guidelines for the Care (European Union Directive (2010/63/EU) and the guidelines for the Use of Laboratory Animals. The procedure was approved by the Committee of Ethics in Research of CIPF. At least eight animals for each group except for histological analysis were used for each type of study (gene expression, GABA content, western blot, and retinal histological quantification).

### Isolation of total RNA and quantitative real-time PCR

Total RNA was isolated from frozen retinas from control and  *rd10* mice at different ages, from postnatal (P) day 15 to P60 (eight retinas for each group) using the NZY Total RNA Isolation Kit (#MB13402, Nzytech, Lisboa, Portugal), following the manufacturer’s protocol. RNA concentration was determined by spectrophotometry on the NanoDrop 2000 (Thermo Fisher Scientific, Wilmington, DE, USA). Then, cDNA was synthesized starting from 200-250 ng of RNA by reverse transcription using the PrimeScript™ RT Reagent Kit (Perfect Real Time) (#RR037A, Takara-Bio, Otsu, Japan), following the manufacturer’s instructions. The cycling conditions consisted of reverse transcription at 37 °C for 15 min and inactivation of reverse transcriptase at 85 °C for five seconds.

The relative expression of *Gabra1*, *Gabre*, *Glra2*, *Slc12a5,* and *Grin1* was measured in retinas by real-time PCR using a thermal cycler (LightCycler® 480 System; Roche, Basel, Switzerland). For the TaqMan gene expression assay, the specific TaqMan probes were: Mm00439046_m1 (*Gabra1*), Mm00489935_m1 (*Gabre*), Mm01168376_m1 (*Glra2*), Mm00803929_m1 (*Slc12a5*), and Mm00433790_m1 (*Grin1*). We used the Premix Ex Taq master mix for probe-based, real-time PCR (#RR390A, Takara-Bio, Otsu, Japan).TATA box binding protein *(Tbp*) gene (Mm00446973_m1) was used as the housekeeping gene. Real-time PCR was performed with one cycle of denaturation of 30 s at 95 °C, continued by 40 cycles of five seconds of denaturation at 95 °C, 30 s annealing at 60 °C, and one cycle of extension at 50 °C for 30 s. Relative gene expression was normalized with the housekeeping gene. Then, normalized values of control mice were normalized to one to determine the changes in the gene expression in *rd10* mice.

### Western blot

Retinas from control and *rd10* mice from P15 to P60 were homogenized in 100 μL of RIPA buffer (20mM Tris-HCl [pH 7.4], 150mM NaCl, 0.1% SDS, 1% Triton X100, and 1mM PMSF) with protease cocktail inhibitor COMPLETE (Roche, Basel, Switzerland)), rested on ice for 5 minutes and sonicated for 20 seconds with a Microson ultrasonic cell disruptor (Misonix Inc, New York, USA) before appropriate dilution in 1× SDS buffer. The protein concentration was measured using the bicinchoninic acid (BCA) protein assay (#23225, BCA Kit; Pierce Scientific, CA, USA), the protein concentration was measured at 550 nm in a Multiskan SkyHigh Microplate Spectrophotometer (Thermo Fisher Scientific, Madrid, Spain). β-mercaptoethanol (5%) (#M7154, Sigma Aldrich, Madrid, Spain) and bromophenol blue (1% wt/vol) (#B0126, Sigma Aldrich, Madrid, Spain) were added to samples. Then, samples were boiled for 5 minutes and electrophoresed on 8% SDS polyacrylamide denaturing gel at 25 mA for two hours, followed by transfer to PVDF membranes (#10600023, Amersham TM, Little Chalfont, United Kingdom) at 250mA for 1.5-2 hours at 4ºC using Mini Trans-Blot Electrophoretic Transfer Cell (#1703930 Bio-Rad, Madrid, Spain). Blots were blocked in 5% BSA and incubated with primary antibody against GABARαRA1 (1:1000, #G4416, Sigma Aldrich, Madrid, Spain), K+ /Cl- cotransporter (KCC2) (1:1000, #07-432, Millipore, Temecula, USA) and NR1 subunit (1:50, #ab109182, Abcam, Cambridge, UK) overnight at 4°C or with peroxidase-conjugated β- tubulin antibody for two hours at room temperature (1:10.000, #AC030, ABClonal, Woburn, USA) under shaking. To detect GABARα1 or KCC2 blots were incubated with a secondary anti-rabbit peroxidase-conjugated antibody (1:10.000, #A6165, Sigma-Aldrich, Madrid, Spain) for an hour at room temperature. Protein bands were detected using NZY Advanced ECL (#MB40201, Nzytech, Lisboa, Portugal) and imaged using the Alliance Q9 Advanced (Uvitec Cambridge, United Kingdom). Western blots were quantified and normalized with β-tubulin using AlphaImager 2200 (alpha innotec, Germany). Twelve retinas were used for each group.

Retinal histology and quantification

Eyes from control and *rd10* mice at P23 (four eyes from each group) were processed for histological analysis. Briefly, the eyes were rapidly removed and fixed in 4% filtered paraformaldehyde (PFA) (#158127, Sigma-Aldrich, Madrid, Spain) for two hours at room temperature and cryoprotected in a sucrose gradient (15–20–30%). Then they were frozen, embedded in Tissue-Tek® O.C.T.™ (Optimal cutting temperature) Compound (Sakura Finetek, Barcelona, Spain) and cut in 10 μm cryosections (Leica CM1900, Nussloch, Germany). Cryosections were post-fixed in 4% filtered PFA pH 7.4 for 15 minutes at room temperature. Cryosections were incubated in a blocking solution containing 3% normal goat serum, 2% bovine serum albumin and 0.25% Triton X-100 (#A1388, Panreac Applichem, Darmstadt, Germany) or a blocking solution containing 5% bovine serum albumin and 0.1% Triton X-100 for one hour. They were later incubated with primary antibody against the glutamate ionotropic receptor NMDA type subunit 1 or NR1 subunit (1:50, #ab109182, Abcam, Cambridge, UK), KCC2 (1:300, #MABN88, Merck Millipore, Darmstadt, Germany), and GABARα1 (1:2000, #G4416, Merck Millipore, Darmstadt, Germany) overnight at 4°C. Then cryosections were incubated with the fluorescence-conjugated secondary antibodies Alexa Fluor 488 or 647 (1:400, #A-11001, #A-21235, Invitrogen, Life Technologies, Madrid, Spain) for one hour at room temperature. After labelling and counterstaining with DAPI (#D9542, Sigma-Aldrich, Madrid, Spain), the sections were mounted in Fluoromount-G (#0100-01, Southern Biotechnology, Birmingham, AL, USA), and observed under a confocal microscope (Leica TCS SP8 MP, Leica Microsystems S.L.U., L’Hospitalet de Llobregat, Spain) using a spatial resolution of 1024 × 1024. The pinhole was set at 1 airy unit and z-stacks were made of 5 pictures (2.0 μm steps) for retinal sections. Stacks were taken under 20x o 40× magnification, with an acquisition rate of 16 frames per second. A zoom factor of 3.0 was employed in some retinal sections. Leica LAX was used as microscope imaging software (Leica Microsystems CMS GmbH, Mannheim, Germany). The integrated densities of GABARα1, KCC2 and NR1 subunit from OPL to GCL (inner retina) were carried out using the ImageJ open-source Software (version 1.53, ImageJ, U.S. National Institutes of Health, Bethesda, Maryland, USA). Adobe Photoshop 10 software (Adobe Systems Inc, San Jose, CA, United States) was used to process the images. To confirm photoreceptor degeneration the number of nuclei at the outer nuclear layer (ONL) was measured. Because the ONL thickness and the degenerative process in the rd10 model vary in different retinal locations, we performed several counts across the entire retina (from the nasal to the temporal retina) for each mouse. Four left and right retinas were analyzed for each group. Slides without primary antibodies served as negative control.

### Determination of GABA in retinal extracts with Liquid Chromatography–Mass Spectrometry (HPLC–MS)

The concentrations of GABA in the retinal extract of control and *rd10* mice were measured with HPLC–MS at P18 and P23. The retinas were homogenized in 100 µL of H2O (specific water for HPLC) on ice, sonicating twice for 15-20 s each time; 20 µL of trifluoroacetic acid was added in a hood to precipitate the proteins, and they were centrifuged at 16,000× g for 15 min at 4◦C. The supernatant was then transferred to another tube to load the 96-well LC–MS plate with 40 µL of the sample. In the pellet, 100 µL of 0.5M NaOH was added and dissolved, and 5 µL was taken to measure the protein concentration. Chromatography was performed on a high-performance liquid chromatography (HPLC) (LC EXION, AB Sciex, Old Connecticut Path, Framingham, MA, USA) system with an Atlantis HILIC Silica column (3.0 microns i.d., 100 × 2.1 mm) (Waters, Milford, MA, USA). The mobile phase contained 0.1% formic acid in water (A) and 0.1% formic acid in acetonitrile (B). The gradient program was: 90% A 0–1.5 min, 15% A at 1.7 min, 15% A 3 min; 90% at 3.1 min, and 90% at 4.5 min. The flow rate was 0.4 mL/min; the column temperature was 30 ◦C, and the injection volume was 30 µL. The HPLC was coupled to a QTRAP 4500 triple quadrupole mass spectrometer (AB Sciex) equipped with an electrospray ionization (ESI) ion source used in positive ion mode. The conditions were the following: inlet potential 10, curtain gas 20, ungrouping potential 46 V, Collision energy 15 eV, GAS1 40 and GAS2 30, 600 ◦C and 4500 V in multiple-reaction monitoring mode (MRM) with the following transition for the quantification of GABA 104 m/z > 87 m/z (decomposition potential 46 V, collision energy 15 eV). A standard curve of GABA [(11)](https://www.zotero.org/google-docs/?dHaI2f) in LCA was used to determine the concentration of these samples with the Analyst program, 1.6.3. from AB Sciex. Nine retinas were used for each group. GABA concentration was expressed in *n*mol/mg protein.

**Bibliography**

[1. Segal M, Xiao Y. Multivariate random forests. WIREs Data Min Knowl Discov. 2011;1(1):80–7.](https://www.zotero.org/google-docs/?CmyOjx)

[2. Lundberg SM, Lee SI. A unified approach to interpreting model predictions. In: Guyon I, Luxburg UV, Bengio S, Wallach H, Fergus R, Vishwanathan S, et al., editors. Advances in neural information processing systems 30 [Internet]. Curran Associates, Inc.; 2017. p. 4765–74. Available from: http://papers.nips.cc/paper/7062-a-unified-approach-to-interpreting-model-predictions.pdf](https://www.zotero.org/google-docs/?CmyOjx)

[3. Lundberg SM, Erion G, Chen H, DeGrave A, Prutkin JM, Nair B, et al. From local explanations to global understanding with explainable AI for trees. Nat Mach Intell. 2020;2(1):2522–5839.](https://www.zotero.org/google-docs/?CmyOjx)

[4. Nogueira S, Sechidis K, Brown G. On the Stability of Feature Selection Algorithms. J Mach Learn Res. 2018;18(174):1–54.](https://www.zotero.org/google-docs/?CmyOjx)

[5. Esteban-Medina M, Peña-Chilet M, Loucera C, Dopazo J. Exploring the druggable space around the Fanconi anemia pathway using machine learning and mechanistic models. BMC Bioinformatics. 2019 Jul;20(1):370–370.](https://www.zotero.org/google-docs/?CmyOjx)

[6. Loucera C, Esteban-Medina M, Rian K, Falco MM, Dopazo J, Peña-Chilet M. Drug repurposing for COVID-19 using machine learning and mechanistic models of signal transduction circuits related to SARS-CoV-2 infection. Signal Transduct Target Ther. 2020 Dec 11;5(1):1–3.](https://www.zotero.org/google-docs/?CmyOjx)

[7. Olivares-González L, Velasco S, Gallego I, Esteban-Medina M, Puras G, Loucera C, et al. An SPM-Enriched Marine Oil Supplement Shifted Microglia Polarization toward M2, Ameliorating Retinal Degeneration in rd10 Mice. Antioxid Basel Switz. 2022 Dec 30;12(1):98.](https://www.zotero.org/google-docs/?CmyOjx)

[8. Loucera C, Esteban-Medina M, Rian K, Dopazo J, Pena-Chilet M. Supplementary data for “The mechanistic functional landscape of Retinitis Pigmentosa: an ML-driven approach to drug repurposing.” 2023 May 22 [cited 2023 Jun 1]; Available from: https://zenodo.org/record/7969017](https://www.zotero.org/google-docs/?CmyOjx)

[9. Mitchell R, Frank E, Holmes G. GPUTreeShap: massively parallel exact calculation of SHAP scores for tree ensembles. PeerJ Comput Sci. 2022 Apr 5;8:e880.](https://www.zotero.org/google-docs/?CmyOjx)

[10.Veleri S, Lazar CH, Chang B, Sieving PA, Banin E, Swaroop A. Biology and therapy of inherited retinal degenerative disease: insights from mouse models. Dis Model Mech. 2015 Feb;8(2):109–29.](https://www.zotero.org/google-docs/?CmyOjx)

[11.[edited by] Robert E. Lenga. The Sigma-Aldrich library of chemical safety data [Internet]. Ed. 2. [Milwaukee, Wis., USA]: Sigma-Aldrich Corp., [1988] ©1988; 1988 [cited 2023 Jun 1]. 4098 p. Available from:](https://www.zotero.org/google-docs/?CmyOjx) <https://search.library.wisc.edu/catalog/999610513502121>
