# Supplementary figures and images for: The mechanistic functional landscape of retinitis pigmentosa: a machine learning-driven approach to therapeutic target discovery
Source: J Transl Med. 2024 Feb 6;22:139. doi: 10.1186/s12967-024-04911-7 (PMC10848380; doi:10.1186/s12967-024-04911-7)

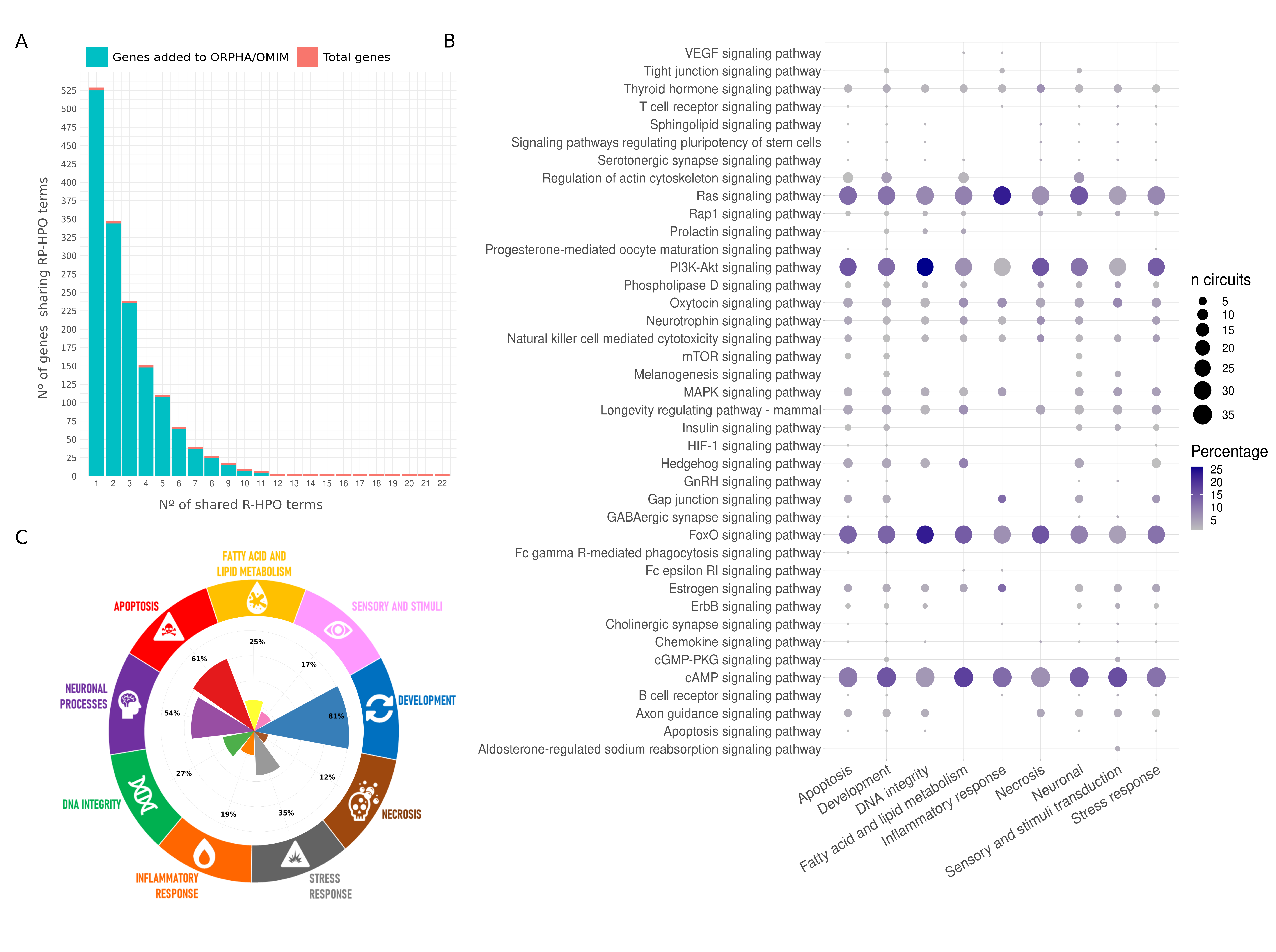

Supplement: Supplementary file 11 — Additional file 11: Figure S1. Composition and functional description of the Retinitis Pigmentosa Map. A) Stacked bar-plot representing the number of genes added to the RP-genes found in ORPHANET/OMIM (blue) from the total number of genes sharing RP-HPO terms (red) in signaling pathways (Y-axis) vs the number of shared RP-HPO terms (X-axis). B) Balloon plot illustrating the percentage, as a color gradient, of the RP-hallmarks (X-axis) represented in each of the 40 KEGG signaling pathways composing the RP Map (Y-axis). The size of the balloon represents the nº of circuits of each pathway. C) Radar plot representing the percentage of the RP map (number of circuits with hallmark tagged/total number of circuits) annotated by each hallmark taking into account that a circuit might be tagged by more than one hallmark. [file 12967_2024_4911_MOESM11_ESM.png]

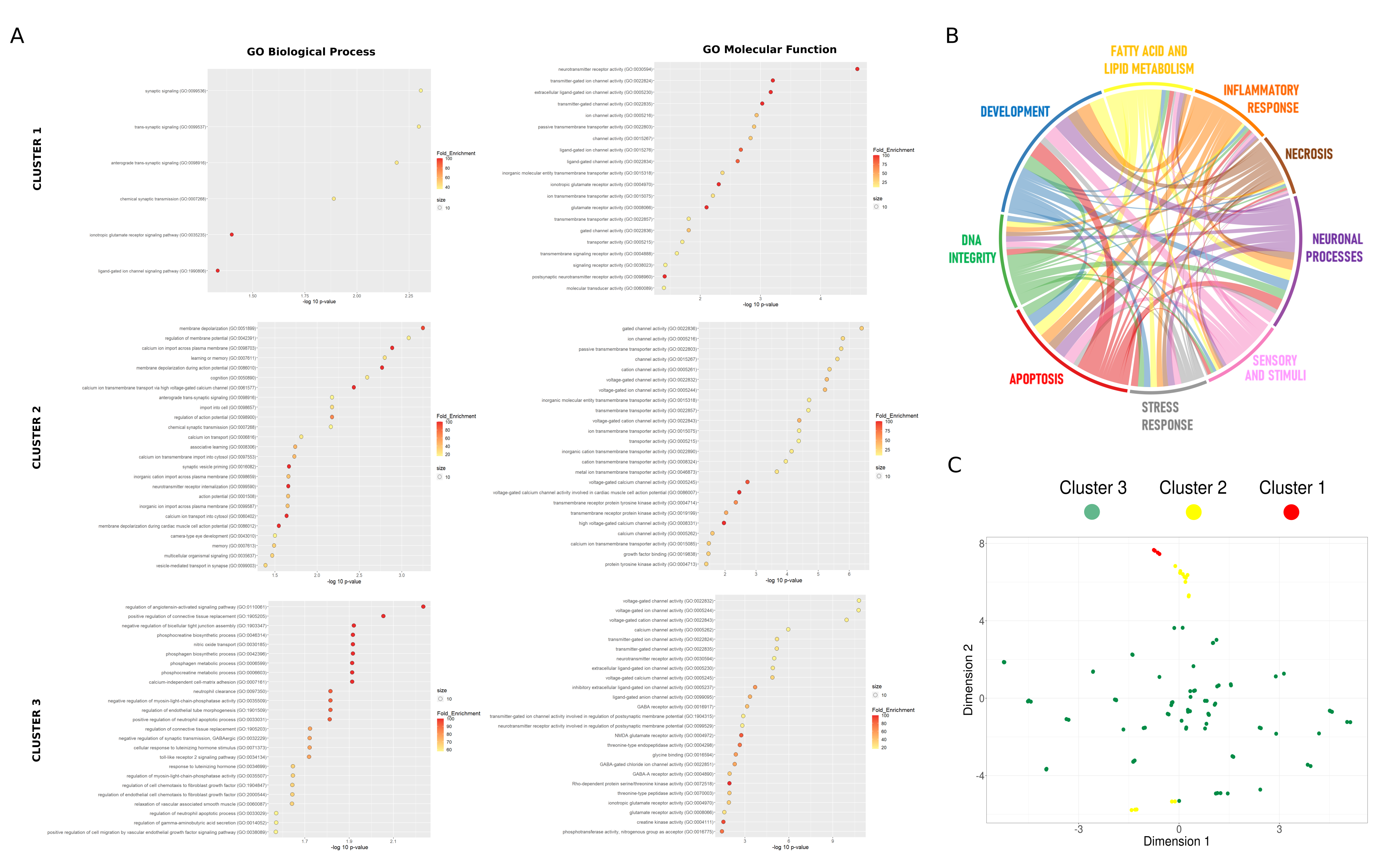

Supplement: Supplementary file 12 — Additional file 12: Figure S2. Profiling and functional analysis of drug target clusters predicted as relevant. A) GAP statistic method metrics for calculating the optimal number of drug target (KDT) clusters, based on their normalized SHAP scores (Gap Statistics chooses the number of clusters where the biggest jump in within-cluster distance occurred). B) Distribution of the SHAP score values of the 109 relevant KDTs on a t-SNE, colored by cluster. C) Gene Ontology (GO) enrichment analysis of significant GO Biological Process and Molecular function terms obtained for the three KDT clusters (cluster 1 in red, cluster 2 in yellow and cluster 3 in green), colored by Fold enrichment. [file 12967_2024_4911_MOESM12_ESM.png]

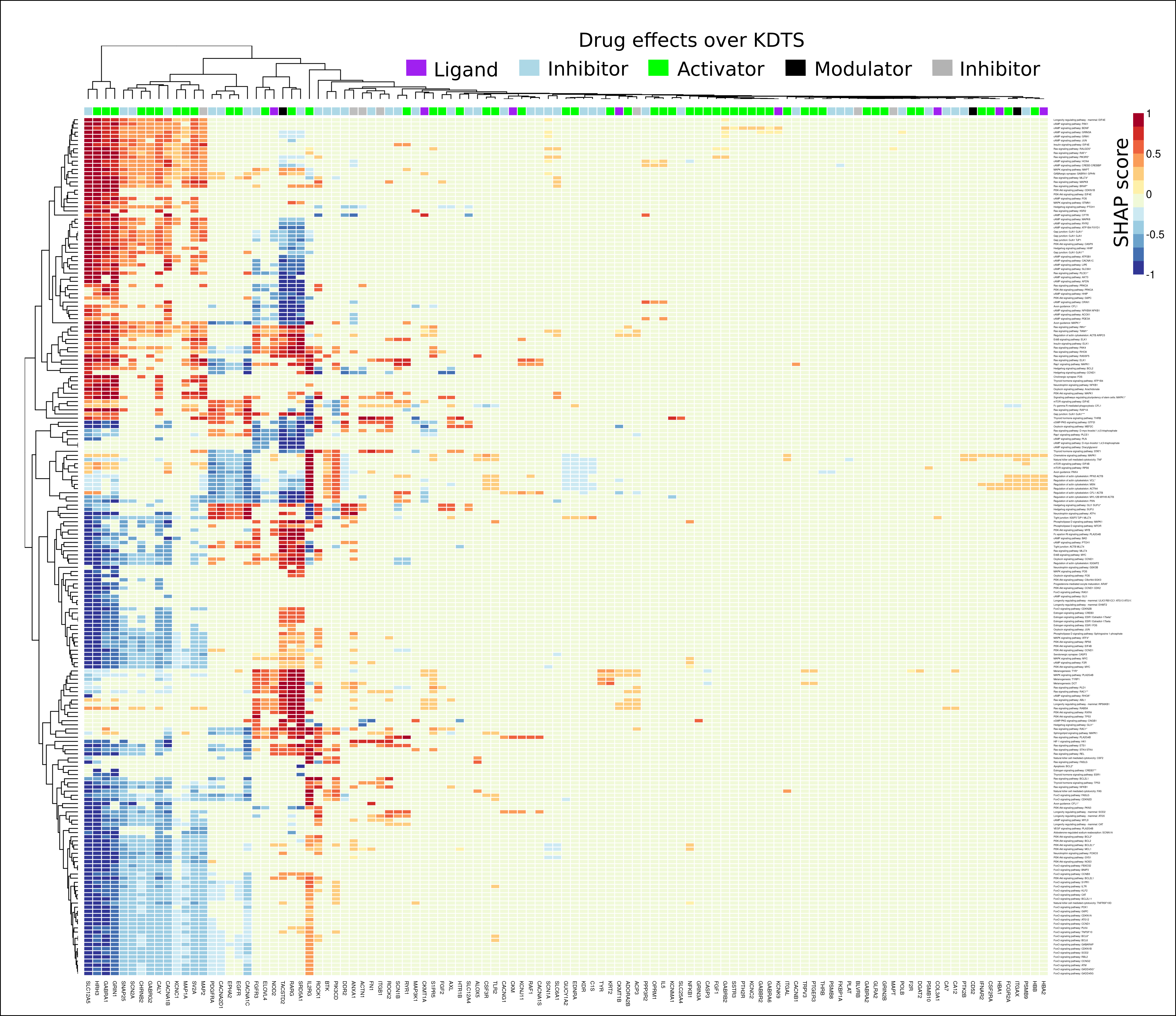

Supplement: Supplementary file 13 — Additional file 13: Figure S3. Heatmap plot of the normalized SHAP scores from the 109 predicted drug targets (KDTs) (X-axis) over the 209 stable circuits (Y-axis) of the Retinitis Pigmentosa Map. The sign indicates the direction of the KDT influence over each specific circuit and the score value depicts how strong is the influence of a specific KDT for predicting the activity of a specific circuit. The top color bar represents the most frequent drug effects of the drugs targeting that specific KDT. [file 12967_2024_4911_MOESM13_ESM.png]

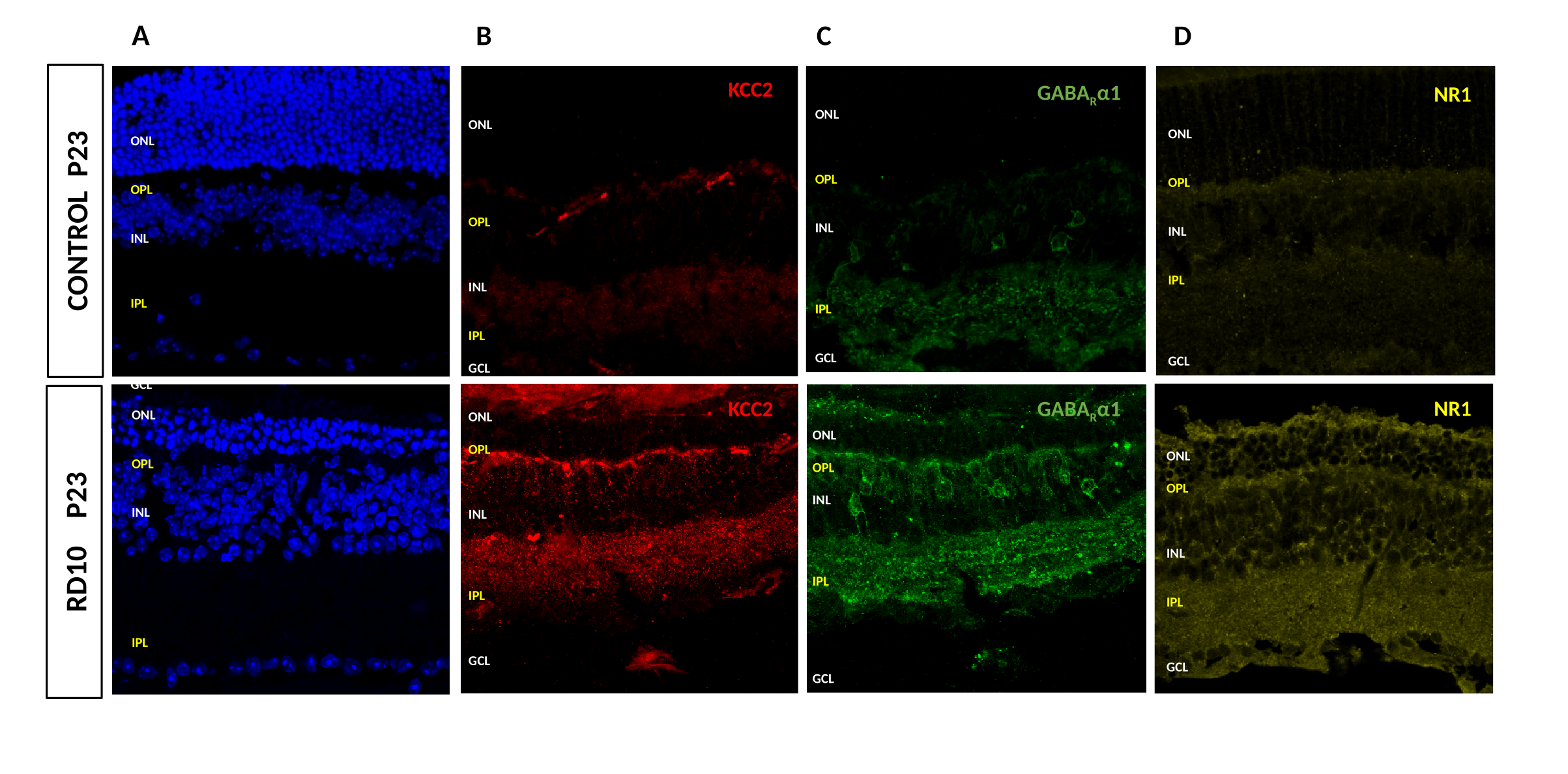

Supplement: Supplementary file 14 — Additional file 14: Figure S4. Augmented histological study of selected KDTs in retinas sections of rd10 mice, murine model of Retinitis Pigmentosa. Confocal micrographs at a 63× augmentations of another set of transverse sections through rd10 mice retina. DAPI nuclear staining illustrating the retinal cytoarchitecture (retinal inner nuclear layer INL, retinal inner plexiform layer IPL, retinal outer nuclear layer ONL, retinal outer plexiform layer OPL and retinal ganglion cell layer GCL). Photoreceptor degeneration (A, B), and retinal distribution of selected KDTs KCC2 (C, D), GABARα1 (E, F) and NR1 (G, H) in control and rd10 mice at P23. [file 12967_2024_4911_MOESM14_ESM.png]
